# Shaping Cell Identity: Global Transcriptome and Pathway Shifts during Mouse Mammary Epithelial Cell Differentiation

**DOI:** 10.34133/csbj.0055

**Published:** 2026-05-04

**Authors:** Waqar Ahmad, Neena Gopinathan Panicker, Tahir A. Rizvi, Farah Mustafa

**Affiliations:** ^1^ Department of Biochemistry and Molecular Biology, College of Medicine & Health Sciences (CMHS), United Arab Emirates (UAE) University, Al Ain, UAE.; ^2^ Department of Microbiology and Immunology, College of Medicine & Health Sciences (CMHS), United Arab Emirates (UAE) University, Al Ain, UAE.; ^3^ Zayed Center for Health Sciences (ZCHS), United Arab Emirates (UAE) University, Al Ain, UAE.

## Abstract

Mouse mammary epithelial cells possess a remarkable ability to regenerate the entire mammary gland through precisely regulated differentiation, involving complex molecular, morphological, and functional changes. Here, we performed comprehensive transcriptomic profiling of HC11 mouse mammary epithelial cells undergoing lactogenic differentiation using RNA sequencing and integrative bioinformatics. We identified 566 differentially expressed genes, reflecting extensive transcriptional reprogramming and activation of biosynthetic, metabolic, and secretory programs. Strong up-regulation of terminal and lactogenic differentiation markers, including Wap, Csn2, Lpl, Cd36, Lalba, Btn1a1, Xdh, Gata3, and Cebpb, signified maturation into a secretory phenotype. Functional evaluation via gene set enrichment analysis revealed transcriptional enrichment of mTOR, prolactin, insulin, ErbB, and autophagy-associated pathways, consistent with anabolic readiness and terminal differentiation. Conversely, p53, Wnt, and FoxO pathways were down-regulated, marking a transition from proliferation to differentiation. Transcription factors (FoxO1, Zbtb16, and Srebf1) and epigenetic regulators (Gadd45a and Hist1h1e) exhibited dynamic changes, underscoring coordinated transcriptional and chromatin remodeling. Gene set enrichment and protein–protein interaction analyses identified 10 hub genes, Agt, Ccnd1, Igf1, Mki67, Myc, Calm4, Rasgrp1, Cd69, Il6, and Pecam1, as central drivers of differentiation. Clustering of uniquely regulated genes further implicated roles in milk synthesis, protease activity, and lineage stabilization. Together, these findings define a transcriptional framework for lactogenic differentiation in the HC11 cell line model and provide a basis for future mechanistic studies.

## Highlights

• Bulk RNA sequencing reveals 566 genes driving lactogenic differentiation in HC11 cells.
• Hormonal (prolactin and insulin), mitogenic (ErbB), and autophagic pathway enrichment was observed.
• Differentiation markers (Wap, Csn2, Lalba, and Gata3) suggest a secretory fate.
• mTOR may act as a central hub integrating hormonal, mitogenic, and autophagic inputs.
• These systems-level insights into lactogenic differentiation need empirical validation.

## Graphical Abstract

**Figure:**
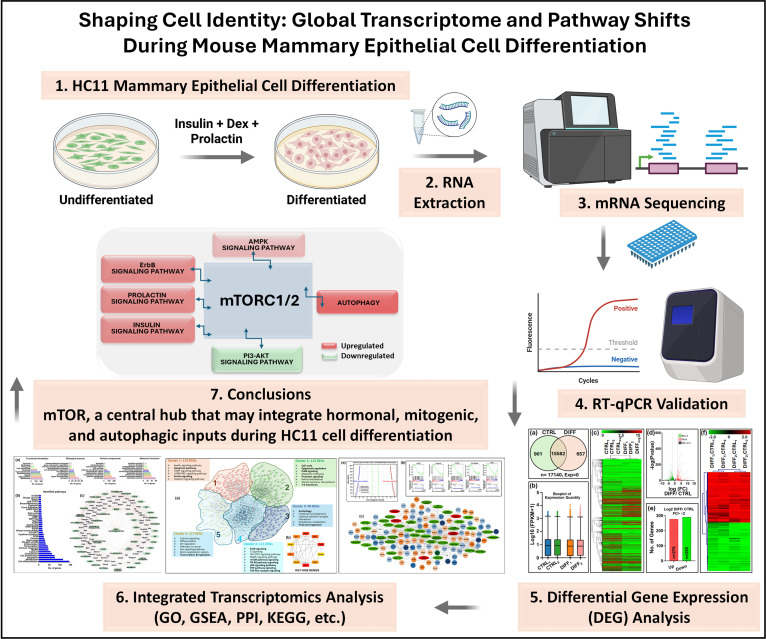

## Introduction

Mammary gland development is a complex and tightly regulated process involving coordinated cellular proliferation, differentiation, and functional maturation to support lactation [1]. In rodents, this transition from cellular proliferation to secretion is most pronounced during pregnancy and lactation, where mammary epithelial cells undergo extensive remodeling to support milk synthesis and its release. This process is governed by dynamic transcriptional programs that regulate key cellular pathways, including milk protein synthesis, hormonal signaling, and extracellular matrix remodeling [1–3]. Understanding the molecular mechanisms underlying this transition is essential for elucidating mammary gland biology and identifying regulatory networks involved in lactogenesis and breast pathophysiology.

The HC11 cell line, derived from midpregnant BALB/c mouse mammary epithelium, has emerged as a robust *in vitro* model for studying differentiation and alveolar maturation. Under specific hormonal treatments like prolactin, dexamethasone, and insulin, these cells adopt a secretory phenotype marked by the induction of milk protein genes, such as *Csn2* and *Wap* [4–7]. While previous studies have characterized select markers of differentiation, a comprehensive transcriptomic analysis, capturing global gene expression changes during this transition, remains limited [2,6–8]. Some earlier transcriptomic studies have shed light on early transcriptional licensing and priming events using glucocorticoid-induced transitions [9–11]; however, the downstream integration of metabolic and biosynthetic pathways governing terminal differentiation remains less well-defined.

In this study, we performed high-throughput RNA sequencing (RNAseq) to profile the transcriptomic landscape of undifferentiated and hormonally differentiated HC11 cells. RNAseq enables unbiased profiling of transcriptomes, capturing low-abundance transcripts, alternative splicing events, and noncoding RNAs with unprecedented sensitivity [12]. Through differential gene expression analysis, gene set enrichment analysis (GSEA), and protein–protein interaction (PPI) mapping, we identify mTOR as a central signaling hub that integrates hormonal (prolactin and insulin), mitogenic (ErbB), and catabolic (autophagy) inputs. Unique gene signatures, such as the up-regulation of Zbtb16, Klk1b family proteases, and casein transcripts, highlight the biosynthetic and structural rewiring essential for full lactogenic commitment. Collectively, these findings provide a systems-level framework for understanding transcriptional reprogramming during lactogenic differentiation in the HC11 cell line model and establish a foundation for future mechanistic studies in mammary epithelial cell biology.

## Materials and Methods

### Cell line and differentiation procedure

Normal BALB/c mouse mammary epithelial cells (HC11) were used to study the effect of differentiation on gene expression. The HC11 cells used in this study were a gift from Prof. Jeffery M. Rosen, Baylor College of Medicine, Houston, TX, USA. The cells were maintained in a complete growth medium containing RPMI 1640 (HyClone, Logan, UT, USA), 10% fetal bovine serum (FBS; HyClone, Logan, UT, USA), 5 μg/ml insulin (Sigma-Aldrich, St. Louis, MI, USA), 0.01 μg/ml epidermal growth factor (EGF; Sigma-Aldrich, St. Louis, MI, USA), and 1% penicillin–streptomycin.

For differentiation, HC11 cells were seeded into 6-well plates at a density of 0.4 × 10^6^ cells per well and grown in the complete growth medium until they reached full confluency. The *in vitro* differentiation protocol was based on a method described earlier [11]. To induce cellular competence, EGF was withdrawn from the medium, and cells were maintained under these conditions for 2 d. This was followed by a 2-d incubation in a predifferentiation medium containing 10% FBS, 5 μg/ml insulin, and 1 × 10^−7^ M dexamethasone. Full differentiation was achieved by treating the cells for 4 additional days with a differentiation medium consisting of 10% FBS, 5 μg/ml insulin, 1 × 10^−7^ M dexamethasone, and 5 μg/ml prolactin. Dexamethasone was obtained from Merck, and natural, pituitary-derived nonrecombinant ovine prolactin (NIDDK-oPRL-21; AFP-10692C) was purchased from the Lundquist Institute for Biomedical Innovation at Harbor-UCLA Medical Center, Torrance, CA, USA. Prolactin was prepared following the supplier’s protocol and stored in desiccated aliquots at −80 °C.

The differentiation of HC11 cells was confirmed through representative assays, as outlined in previously established protocols with minor modifications [13]. Briefly, lipid accumulation, a hallmark of mammary epithelial cell differentiation, was assessed using Oil Red O (ORO) staining. Undifferentiated and differentiated cells were fixed in 10% formalin, permeabilized with 60% isopropanol, and stained with freshly prepared ORO solution. Excess stain was removed with distilled water, and lipid-positive cells were visualized under light microscopy.

In addition, β-casein protein expression analysis was conducted via western blotting using antibodies from Santa Cruz Biotechnology, Inc., Dallas, TX, USA. Cell lysates were extracted using radioimmunoprecipitation assay buffer supplemented with protease inhibitors. Protein concentration was determined using the Bradford assay, and equal amounts (40 μg) were resolved on sodium dodecyl sulfate–polyacrylamide gel electrophoresis, transferred to nitrocellulose membranes, and probed with specific primary and horseradish peroxidase-conjugated secondary antibodies. Detection was performed via chemiluminescence, and β-actin was used as a loading control.

### RNA extraction and mRNA sequencing

Total RNA was extracted from differentiated and undifferentiated HC11 cells using the TRIzol reagent (Thermo Fisher Scientific, Waltham, MA, USA) and quantified using Nano Drop, as described previously [14]. Whole-cell RNA from 2 independent differentiation experiments of HC11 cells was sequenced commercially by the Beijing Genomics Institute (BGI, Hong Kong) at the same time using the TruSeq library and DNBSEQ platform. The RNA integrity number values for the RNA samples ranged between 8.5 and 9.5, as assessed by Agilent 2100 Bioanalyzer. Before sequencing, total RNA was treated with DNase I to remove any contaminating DNA, and the messenger RNA (mRNA) was enriched using oligo(dT) magnetic beads. This was followed by complementary DNA synthesis, end repair, addition of A and adaptor ligation, polymerase chain reaction (PCR), circularization, and sequencing (https://www.bgi.com/global/science-detail/transcriptome-sequencing).

### RNAseq data preprocessing

RNAseq data processing was thoroughly described in our previous studies using different datasets [15–17]. A detailed data analysis pipeline by BGI is provided in Fig. S1. All RNA samples were processed under identical library preparation conditions and sequenced within the same sequencing run to minimize technical variability. Briefly, RNAseq was performed on control and differentiated HC11 total RNA samples from 2 independent experiments, named CTRL1, CTRL2, DIFF1, and DIFF2, respectively, and all samples were processed and sequenced within the same batch at the same time; therefore, no batch correction was applied. SOAPnuke v1.5.2 [18] was used to filter reads of low quality with adaptor contamination, and excessively high levels of unknown base *N* (*N* > 5%). The filtered clean reads were next subjected to quality control (QC) using FastQC [19]. Pearson correlation coefficients were calculated between biological replicates to assess sample consistency. Although BGI’s RNAseq QC documentation does not specify a fixed Pearson correlation cutoff, we considered a threshold of *r* ≥ 0.90 as acceptable for replicate QC. All biological replicates in this study exceeded this cutoff, with within-group correlations above 0.98.

A total of 102.42 million reads (100-bp length) were generated for each sample and subjected to QC. Raw data were cleaned, which limited the read count to 101.6, 100.46, 100.24, and 100.06 million reads for CTRL1, CTRL2, DIFF1, and DIFF2 samples, respectively. The clean reads were aligned to the reference mouse genome sequence GCF_000001635.26_GRCm38.p6 (*Mus musculus* using HISTAT2 v2.0.4) with an average alignment of 94.13% using HISTAT2 [20]. Gene-level quantification resulted in 57,378 transcript entries prior to filtering (Table S1). The reference genes were aligned to the data using Bowtie2 [21]. The average alignment of the gene set was 94.455% for CTRL and 94.06% for DIFF. A total of 17,140 transcripts were detected (Table S2) for both groups. The results were submitted to the BGI in-house software Dr. Tom accessed through an online server for further analysis. The raw data can be downloaded from the National Center for Biotechnology Information BioProject server for data reanalysis and further processing (https://www.ncbi.nlm.nih.gov/bioproject/?term=PRJNA915407).

### Validation of gene expression using quantitative real-time PCR

Although RNAseq provides high sensitivity and a broad dynamic range for transcriptome profiling [22,23], quantitative real-time PCR (RT-qPCR) was performed on selected genes using SYBR Green chemistry to confirm our findings. The same complementary DNA samples derived from undifferentiated and differentiated cells were used as templates. Reactions were set up with HOT FIREPol EvaGreen qPCR Mix (Solis BioDyne, Estonia), and β-actin was used as the endogenous control. The RT-qPCRs were carried out in 96- or 384-well plates in duplicates using prevalidated KiCqStart primers (Merck, Rahway, NJ, USA), as listed in Table S3. Primer specificity was confirmed by single-band amplification and single-peak melt curve profiles. Relative gene expression was calculated using the comparative delta-delta cycle threshold (ΔΔCt) method. Ct values were first normalized to an internal reference gene, β-actin, and fold changes (FCs) or relative quantification values were calculated relative to the undifferentiated control cells. The relative quantification values were then used for plotting and analysis.

### Identification of DEGs

After read cleaning and alignment to the reference genome, differential expression analysis was performed using DESeq2 [24], which applies a negative binomial model with Benjamini–Hochberg false discovery rate (FDR) correction. For primary biological interpretation, high-confidence differentially expressed genes (DEGs) were defined using stringent criteria of FDR-adjusted *P* value ≤ 0.05 and |log_2_ FC| ≥ 1 (equivalent to FC ≥ 2 or FC ≤ 0.5). These genes are referred to as “high-confidence DEGs” throughout the article and are highlighted in red (up-regulated) and green (down-regulated) (Table S2). Genes were considered condition restricted if transcripts per million (TPM) > 1 was observed in both replicates of one condition and TPM = 0 in both replicates of the other condition.

For pathway enrichment and cross-study comparative analyses, a relaxed threshold (|FC| ≥ 1) was applied to retain moderately regulated genes that may contribute to coordinated gene-set-level effects. This approach preserves pathway-level sensitivity and ensures methodological comparability with previously published datasets using similar criteria. All DEG lists were downloaded from Dr. Tom and independently verified for consistency prior to downstream analysis. Venn diagrams were generated using the Bioinformatics & Evolutionary Genomics platform (Van de Peer lab; http://bioinformatics.psb.ugent.be/webtools/Venn/). Heatmaps were generated using Multiple Experiment Viewer v4.9.0 [25]. The volcano and other plots used in this study were generated through Dr. Tom and were further improved accordingly.

### Functional enrichment of Gene Ontology and pathway analysis

DEGs were analyzed for Gene Ontology (GO) and pathway enrichment using the DAVID v6.8 platform [26] (https://davidbioinformatics.nih.gov/tools.jsp) with *M. musculus* as the reference species. GO analysis categorized genes based on biological processes, cellular components, and molecular functions (Table S4). Pathway annotation was primarily conducted using the Kyoto Encyclopedia of Genes and Genomes (KEGG) (https://www.genome.jp/kegg/) [27], Reactome (https://reactome.org/) [28], and WikiPathways databases (https://www.wikipathways.org/) [29]. For DEGs lacking annotations in these databases, additional information was retrieved from Mouse Genome Informatics (https://www.informatics.jax.org/) [30], GeneCards (https://www.genecards.org/) [31], and the Rat Genome Database for mouse (https://rgd.mcw.edu/wg/species/mouse/) [32]. Genes without functional or pathway information were classified as “uncharacterized”.

To identify transcription factors (TFs) and epigenetic regulators (ERs) among the DEGs, we queried the Transcriptional Regulatory Relationships Unraveled by Sentence-based Text mining (TRRUST) (https://www.grnpedia.org/trrust/) [33] and EpiFactors (https://epifactors.autosome.org/) databases [34]. Since EpiFactors is human centric, identified genes were filtered for mouse-specific entries using DAVID after species selection. Additional annotations and network data were supplemented using STRING v11.5 (https://string-db.org/) [35] and the Dr. Tom online module (https://biosys.bgi.com/) when needed. Final gene lists used for downstream analysis were curated based on cross-validation across all these resources.

### Gene set/pathway enrichment analysis

Gene Set Enrichment Analysis (GSEA) v4.10 [36] was used to evaluate the possibility of gene set associations with specific phenotypes. GSEA was performed using expression sets of whole genes in a pathway rather than DEGs. The gene sets were retrieved from the KEGG database for individual pathways. The GSEA run used the following parameters: 1,000 permutations, “weighted” as enrichment statistic, and “Signal2Noise ratio” as a metric for ranking the genes. The gene sets having an FDR ≤ 0.025 were selected as significant (default) and were further filtered down by selecting the sets having family-wise error rate (FWER) *P* value ≤ 0.2 (the probability of making one or more false discoveries).

### Construction of the gene–pathway, PPI, and hub gene networks

The significant pathways were interrogated for overlapping and unique DEGs representing individual pathways. The online database STRING v11.5 was used to construct PPI networks, while Cytoscape v3.10.3 [37] was used to view these networks and to construct gene–pathway interactions networks. CytoHubba [38], a plugin of Cytoscape, was used to detect the hub genes from network analysis using the maximum clique centrality algorithm, whereas MCODE [39] was used to identify any significant module for PPI networks.

## Results

**Fig. 1.**
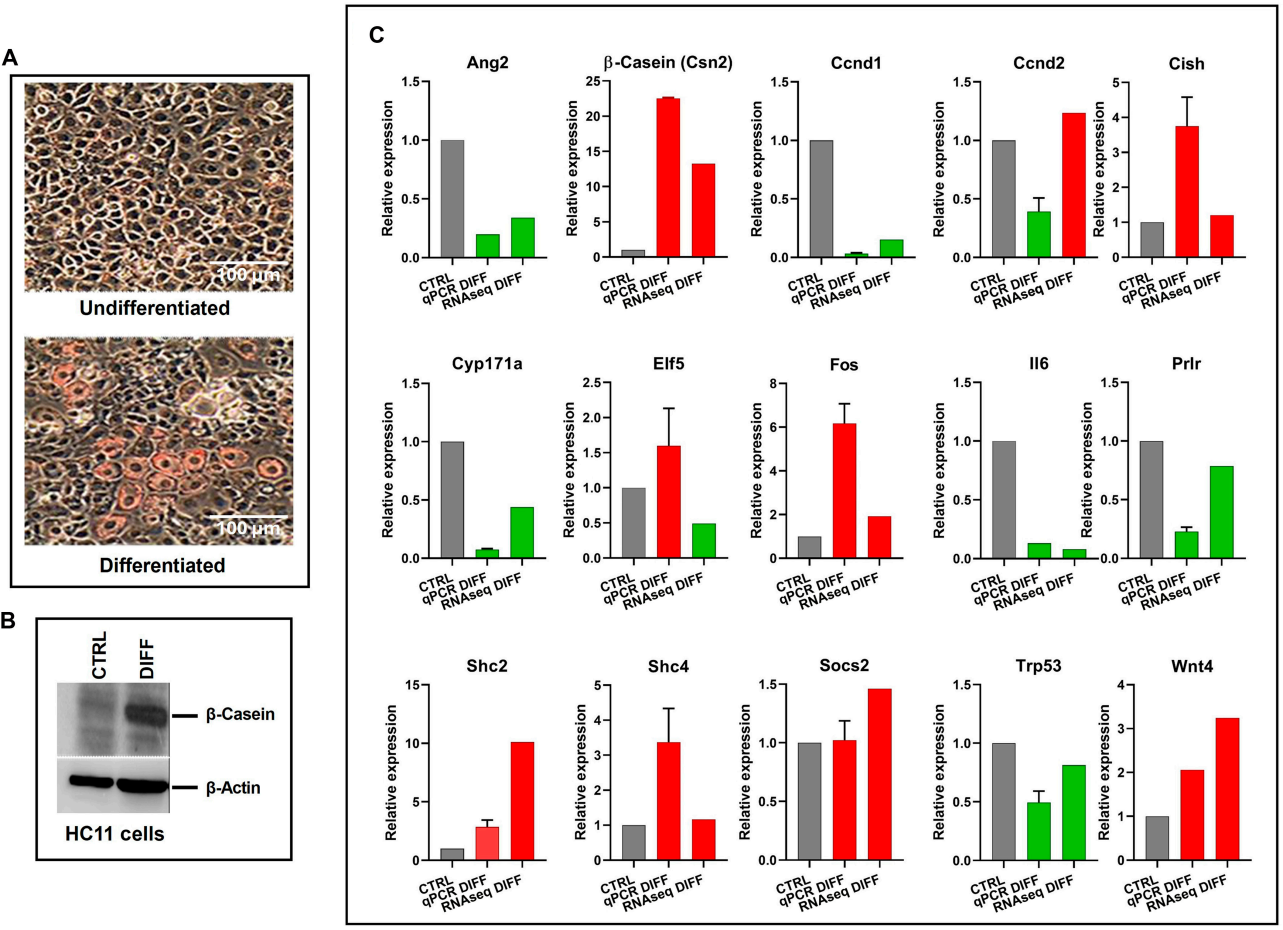
Validation of differentiation and RNA sequencing (RNAseq) gene expression by quantitative real-time polymerase chain reaction (RT-qPCR) in HC11 mammary epithelial cells before and after differentiation. (A) Oil Red O staining of undifferentiated and differentiated HC11 cells for lipid droplets. Representative micrograph of HC11 cells (40× objective). The scale bar represents 100 μm. (B) Representative anti-β-casein antibody western blotting showing induced β-casein in HC11 differentiated cells (DIFF) when compared with undifferentiated cells (CTRL). (C) Relative expression levels of 15 selected genes were analyzed by RT-qPCR (middle bar in each graph) and compared with RNAseq data (right bar in each graph). The expression of each gene in undifferentiated cells (CTRL, left bar, gray) was set to 1. The consistency in directional change between RNAseq and RT-qPCR data confirms the reliability of transcriptomic profiling. Error bars represent standard deviation between biological replicates. Red bars represent up-regulation of gene expression, while green bars represent down-regulation of gene expression compared to that in control HC11 cells.

HC11 mouse mammary epithelial cells were differentiated using established protocols. To validate successful differentiation, first we performed ORO staining and western blot analysis using an anti-β-casein antibody. ORO staining, used as an indicator of intracellular lipid accumulation associated with milk synthesis, revealed red-stained lipid droplets (Fig. 1A), while western blotting demonstrated induction of β-casein protein expression (Fig. 1B), both recognized hallmarks of mammary epithelial cell differentiation. However, ORO staining exhibited heterogeneity across the cell population, suggesting that differentiation was not entirely uniform. The total RNA isolated from the control (CTRL) and differentiated (DIFF) cultures was used to conduct comparative RNAseq analysis using 2 independently generated biological replicates. Accordingly, the RNAseq data represent the average transcriptional profile of a differentiation-enriched population (bulk RNAseq) rather than a fully homogeneous cell state. To minimize this shortcoming, comparisons were made relative to undifferentiated controls, ensuring that observed transcriptional changes reflected differentiation-associated programs, while any residual population heterogeneity would be similarly represented across replicates and not confound the relative analysis.

To confirm the RNAseq data, we performed RT-qPCR on a subset of genes involved in mammary epithelial cell differentiation (β-casein, Elf5, Cish, Fos, and Wnt4), cell-cycle regulators (Ccnd1 and Ccnd2), cytokine signaling genes (Il6, Prlr, and Socs2), and TFs (Trp53, Shc2, and Shc4). Undifferentiated HC11 cells were used as the baseline (expression set to 1), and gene expression changes upon differentiation were compared across RT-qPCR and RNAseq platforms (Fig. 1C). The validation results demonstrated strong concordance in expression trends for the majority of genes, including up-regulation of β-casein, Cish, Fos, and Wnt4, key regulators associated with mammary gland maturation. Likewise, down-regulation of Ccnd1 and Il6 further supported the differentiation phenotype. Comparison of FC values obtained by RNAseq and RT-qPCR across 15 validated genes revealed strong overall concordance (Spearman *ρ* = 0.82, *P* < 0.001). Although minor discrepancies in magnitude were observed for a small subset of genes, the majority displayed consistent directional regulation, supporting the reliability of transcriptomic findings. Bland–Altman analysis further demonstrated acceptable agreement between methods, with most genes falling within the 95% limits of agreement and a modest mean difference of −0.55, indicating slightly lower FC estimates by RNAseq relative to RT-qPCR (Table S5). Larger deviations were primarily associated with genes exhibiting high FCs, consistent with known differences in dynamic range and normalization between platforms. Collectively, these analyses support the overall reliability and cross-platform consistency of the transcriptomic findings.

### Identification of DEGs in differentiated mammary epithelial cells

**Fig. 2.**
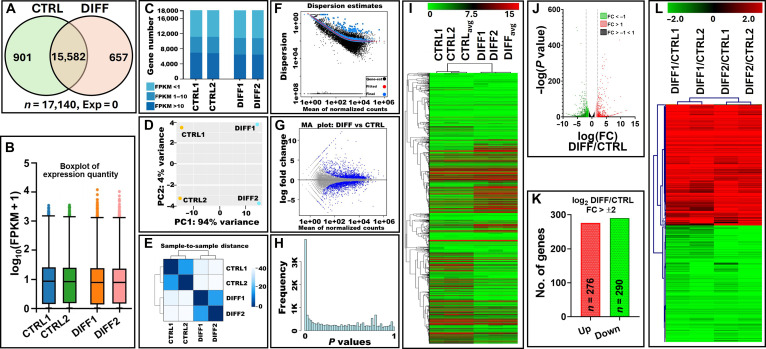
Comprehensive overview of RNA sequencing (RNAseq) quality control, expression distribution, and differential gene expression analysis between undifferentiated (CTRL) and differentiated (DIFF) HC11 cells. Two independent biological replicates per condition (CTRL1, CTRL2, DIFF1, and DIFF2) were sequenced and analyzed using the Dr. Tom (Beijing Genomics Institute [BGI]) platform and DESeq2. A cross-sectional comparison was made between the groups to identify differences. (A) Venn diagram showing the distribution of detected transcripts between CTRL and DIFF groups. (B) Tukey boxplots of normalized gene expression values as fragments per kilobase of exon per million mapped reads (log_10_ FPKM + 1) across all 4 replicates, demonstrating comparable expression distributions and consistent median values among samples. (C) Stacked bar plot of gene abundance distribution across FPKM categories (FPKM ≤1, 1 to 10, and ≥10). (D) Principal component analysis (PCA) of normalized count data. (E) Sample-to-sample distance heatmap with hierarchical clustering. (F) Dispersion estimates from DESeq2 analysis, showing gene-wise dispersion (black), fitted dispersion trend (red), and final shrinkage-adjusted dispersion values (blue). (G) Minus-average plot (MA plot) of differential gene expression (DIFF vs CTRL), displaying log_2_ fold change (FC) versus mean normalized counts. (H) Histogram of the raw *P* value distribution from differential expression testing. (I) Hierarchical clustering heatmap of all expressed genes, illustrating global transcriptional patterns and separation between CTRL and DIFF samples. (J) The scatter plot of differentially expressed genes (DEGs) with FC > ±1 highlights the expression profile of DEGs and non-DEGs. Red dots represent up-regulated genes, green dots indicate down-regulated genes, and gray dots denote non-DEGs. (K) Mapping of DEGs reveals the up-regulated (red) and down-regulated genes (green) in each comparison included in this study, defined by FC ≥ 2. (L) A heatmap displaying the hierarchical clustering of 566 DEGs (FC ≥ 2) illustrates distinct expression patterns among all groups.

RNAseq analysis revealed a total of 17,140 transcripts across all samples. Of these, 15,582 transcripts were shared between the CTRL and HC11 DIFF samples, while 901 transcripts were unique to CTRL and 657 to DIFF (Fig. 2A and Table S2). Global expression distributions were first assessed to evaluate data quality and comparability between samples. Boxplot analysis of normalized expression values as fragments per kilobase of exon per million mapped reads (log_10_ FPKM + 1) demonstrated highly consistent median expression levels and interquartile ranges across all 4 biological replicates (CTRL1, CTRL2, DIFF1, and DIFF2), indicating uniform library complexity and sequencing depth (Fig. 2B). Similarly, stacked bar plots categorizing genes by expression abundance (FPKM ≤1, 1 to 10, and ≥10) revealed comparable transcriptome complexity across samples, suggesting that differentiation does not globally alter transcript abundance distribution but instead induces targeted transcriptional changes (Fig. 2C).

To further evaluate sample relationships and reproducibility, principal component analysis was performed using normalized count data. Principal component analysis demonstrated clear separation between CTRL and DIFF samples along PC1, which accounted for 94% of total variance, while PC2 accounted for 4% (Fig. 2D). Biological replicates clustered closely within each condition, indicating high reproducibility and minimal batch variation. Consistently, hierarchical clustering based on sample-to-sample distance metrics confirmed tight clustering of replicates within groups and distinct separation between undifferentiated and differentiated states (Fig. 2E), revealing the samples clustered according to biological condition rather than technical factors.

Variance modeling and statistical robustness were assessed using DESeq2. Dispersion estimates exhibited the expected inverse relationship between mean normalized counts and dispersion values, with gene-wise estimates appropriately shrunk toward the fitted dispersion trend, confirming stable variance estimation (Fig. 2F). The minus-average plot further demonstrated symmetric distribution of log_2_ FCs around zero, with significantly DEGs highlighted and no evidence of systematic expression bias (Fig. 2G). Additionally, the histogram of raw *P* values showed enrichment near zero, consistent with the presence of true biological signals rather than random noise (Fig. 2H).

As mentioned earlier, 2 independent biological replicates were analyzed per condition. While replicate concordance and high sequencing depth support robust detection of major transcriptional differences, the limited number of biological replicates reduces the statistical power for detecting subtle expression changes. Therefore, the results should be interpreted as reflecting large-effect transcriptional shifts associated with HC11 differentiation [40]. The gene expression analysis in our study demonstrated consistency and reliability, further supported by the visualization of DEGs using a heatmap. The heatmap highlighted distinct patterns of up-regulated and down-regulated genes between the groups (Fig. 2I), providing clear insights into the transcriptional changes associated with HC11 differentiation. Hierarchical clustering confirmed consistent data distribution within each subgroup, further validating the reliability of the dataset. Unique transcripts within each group were excluded, and the remaining 15,582 shared transcripts were analyzed to identify DEGs using the DESeq2 method. This was achieved by using stringent filtering criteria (*P* value < 0.05 and *Q* value < 0.1), with manual verification, confirming the results. The scatter plot demonstrated a normal distribution of read counts, with significantly altered genes highlighted as DEGs (Fig. 2J). In total, 1,522 genes were differentially expressed with FC > ±1, including 635 up-regulated and 887 down-regulated transcripts. Of these, 566 DEGs exhibited FC > ±2, comprising 276 up-regulated (48.7%) and 290 down-regulated (51.3%) genes (Fig. 2K), indicating a slight predominance of transcriptional repression during differentiation. Expression consistency across the duplicate biological samples was further confirmed in Fig. 2L. Thus, overall, hierarchical clustering combined with stringent statistical filtering and manual validation supported the robustness, reproducibility, and biological relevance of our RNAseq data, providing a solid foundation for downstream functional and pathway analyses.

### GO analysis of the DEGs

**Fig. 3.**
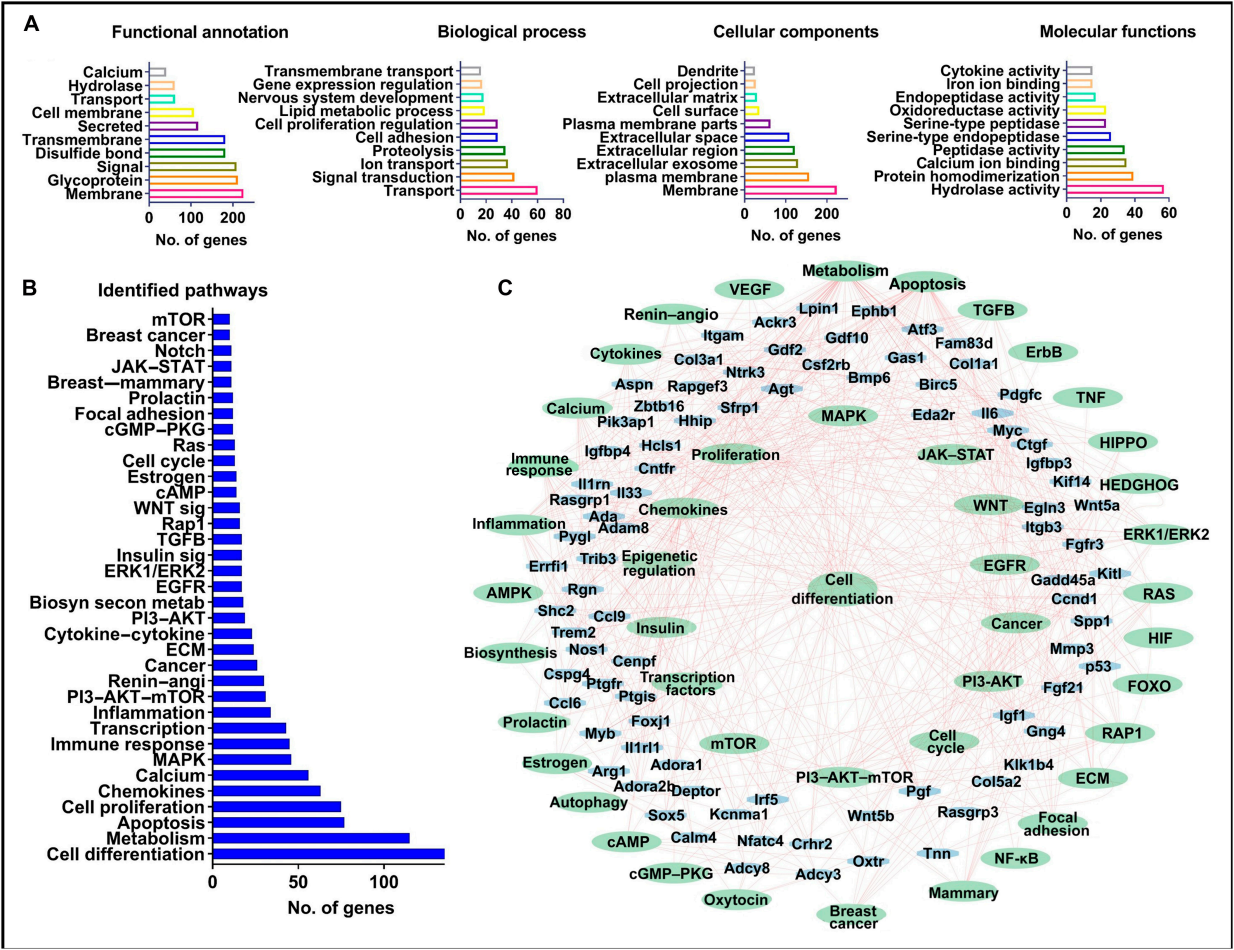
Gene Ontology (GO) analysis and gene–pathway interaction network associated with cellular differentiation of the differentially expressed genes (DEGs). (A) Classification of the DEGs based on their functional annotation, biological processes, cellular locations, and molecular functions. The top 10 subcategories based on the number DEGs in each category are represented in each graph. (B) Biological pathway classification of DEGs. The DEGs were grouped in different pathways based on the information gathered from different online pathways sources, including Kyoto Encyclopedia of Genes and Genomes (KEGG), WikiPathways, and/or Reactome. Each bar shows the number of genes associated with the subsequent pathway. (C) The network depicts the interaction between genes (light blue ovals) and key biological pathways (green ovals) involved in cellular differentiation.

GO analysis was performed to explore the functional, biological, cellular, and molecular attributes of the DEGs. Based on the accumulated information from all sources mentioned in the materials and methods section, each gene was categorized according to its known function or pathway association. Figure 3 highlights the top 10 annotations for each category based on the number of genes in each set; additional annotations are provided in Table S4. Functional annotation analysis of the DEGs identified key roles in cell differentiation, with the majority of genes associated with the cell membrane, transmembrane domains, transportation, glycoproteins, and signaling pathways. Biological process analysis revealed that the DEGs were predominantly involved in transmembrane transport, gene expression regulation, lipid metabolic processes, cell proliferation regulation, proteolysis, and signal transduction (Fig. 3A). These processes are critical for orchestrating cellular differentiation and maintaining tissue-specific functionality. Cellular localization analysis indicated that most DEGs were localized to the cellular membrane, extracellular space, and plasma membrane, emphasizing their roles in cell–environment interactions essential for differentiation. Molecular functional characterization further demonstrated the involvement of DEGs in cytokine activity, hydrolytic processes, metal ion binding, peptidase activity, and oxidoreductase activity, highlighting their contributions to the biochemical and signaling pathways underlying differentiation (Fig. 3A).

To further explore the biological relevance of the DEGs, we investigated their involvement in key biological pathways. Figure 3B summarizes the accumulated information for each gene, derived from various data sources, as previously described [27–29,33]. Notably, many of the DEGs were found to overlap across multiple pathways (Table S6), highlighting their diverse roles in cellular processes. Pathway analysis revealed several signaling pathways significantly associated with the DEGs, including those involved in metabolism, apoptosis, cell proliferation, and various signal transduction cascades. These pathways included MAPK signaling, immune response, transcriptional regulation, PI3–AKT–mTOR signaling, the renin–angiotensin system, EGFR signaling, insulin signaling, Rap1, Wnt, cAMP, estrogen signaling, cell-cycle regulation, focal adhesion, prolactin signaling, Notch signaling, and mTOR signaling (Fig. 3B). These pathways are crucial for the regulation of cell differentiation, proliferation, and survival, underscoring their importance in the molecular mechanisms driving cellular differentiation.

To identify biological processes associated with cellular differentiation, we constructed a gene–pathway interaction network integrating DEGs with enriched functional pathways. The resulting network (Fig. 3C) revealed several major signaling hubs, including MAPK, WNT, PI3K–AKT, TGF-β, and mTOR, which were densely connected to a large number of genes, suggesting their central role in coordinating differentiation. Notably, genes such as Ccnd1, Sox5, Bmp6, Wnt5a, and FoxO3 served as critical nodes bridging multiple pathways. In addition to classical differentiation-related pathways, the network highlighted the involvement of immune response, inflammatory signaling (NF-κB, TNF, etc.), and ERs, indicating broader regulatory mechanisms. Pathways associated with hormonal signaling (e.g., estrogen, prolactin, and oxytocin) and cancer-related processes (e.g., ERK1/ERK2, apoptosis, and breast cancer) were also enriched, suggesting potential links between differentiation and disease-relevant transcriptional programs. The integration of pathway enrichment and gene interaction data enabled a comprehensive view of the molecular networks underlying differentiation, emphasizing the multilayered regulation involving signal transduction, transcriptional control, metabolic reprogramming, and chromatin remodeling.

### PPI network of DEGs regulated during differentiation

**Fig. 4.**
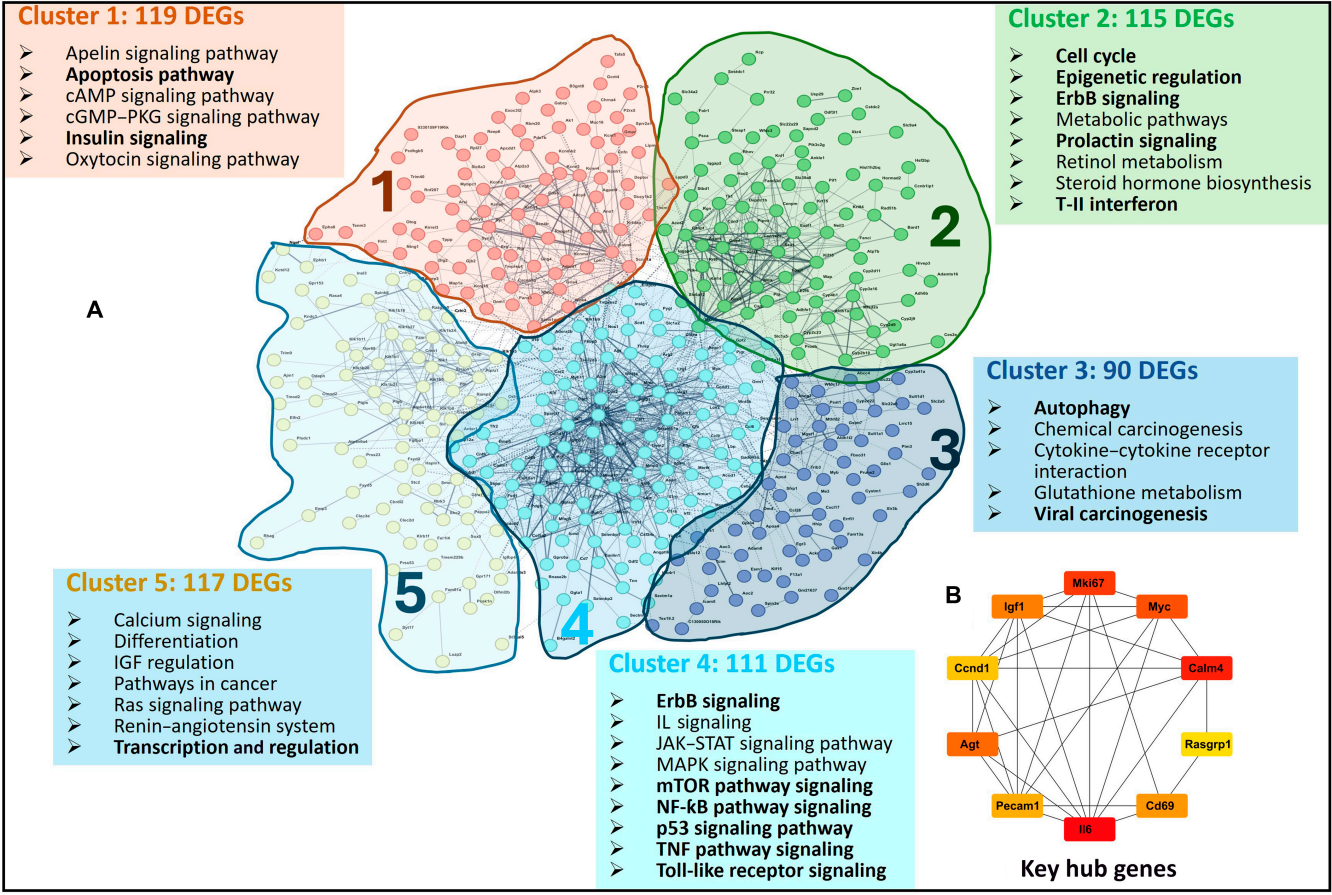
Protein–protein interaction (PPI) analysis and clustering of differentially expressed genes (DEGs). STRING was used to create the PPI network using default settings and selecting *Mus musculus* as the reference species. (A) PPI network analysis using STRING showing that out of 566 DEGs, 552 can be further divided into 5 subgroups based on their biological functions. (B) The top 10 hub genes identified using the CytoHubba module in Cytoscape.

To better understand the regulatory mechanisms associated with HC11 differentiation, DEGs were used to construct PPI networks through the STRING interactome database. The analysis of all DEGs identified 5 distinct sub-networks using the *k*-means clustering method, grouped based on their functional attributes (Fig. 4A). These clusters were further analyzed and found to be associated with several key pathways critical to cellular differentiation, including autophagy, apoptosis, insulin signaling, cell-cycle regulation, ErbB signaling, epigenetic regulation, transcriptional control, JAK–STAT signaling, NF-κB signaling, and the mTOR pathway. STRING proved to be a powerful tool in identifying functional modules, enriched pathways, and significant hub genes within the networks. Hub genes, representing highly connected nodes within the PPI network, play pivotal roles as key regulators or drivers of phenotypic changes. These genes are often central to maintaining the structural and functional integrity of the network. To identify such genes, the CytoHubba plugin in Cytoscape was employed, generating a ranked list of hub genes based on the STRING-derived PPI network (Fig. 4B). In this study, 10 hub genes were identified as potential key regulators: Agt, Ccnd1, Igf1, Mki67, Myc, Calm4, Rasgrap1, Cd69, Il6, and Pecam1. These genes are involved in diverse cellular processes, including proliferation, differentiation, apoptosis, cell-cycle progression, cell signaling, and molecular transport.

### Gene enrichment analysis of the DEGs associated with different pathways during differentiation

Following DEG identification, GO profiling, and STRING analysis, GSEA was performed to evaluate coordinated transcriptional changes at the pathway level during HC11 differentiation. GSEA assesses whether predefined gene sets are statistically overrepresented at the extremes of a ranked gene list. It calculates an enrichment score to determine whether a gene set is overrepresented at the top or bottom of a ranked gene list. The normalized enrichment score accounts for gene set size and variation across phenotypes, and significant gene sets were selected based on FDR < 0.25 and FWER *P* value < 0.2 (https://www.gsea-msigdb.org/gsea/index.jsp) [36]. Importantly, GSEA identifies coordinated transcriptional enrichment of pathway-associated genes and does not directly measure functional pathway activation.

**Fig. 5.**
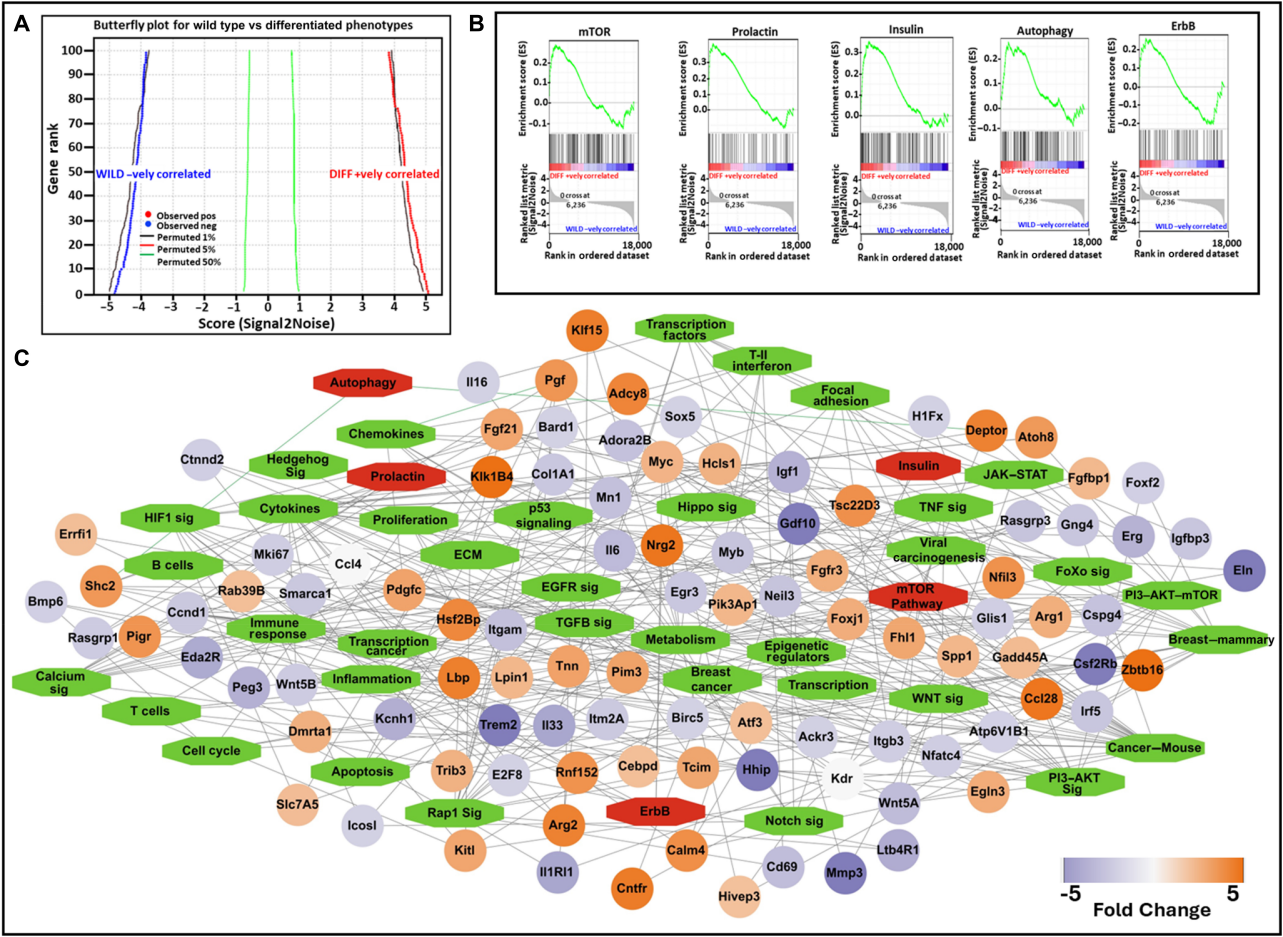
Networking and gene set enrichment analysis (GSEA) for the differentially expressed genes (DEGs) associated with HC11 differentiation. A total of 56 Kyoto Encyclopedia of Genes and Genomes (KEGG) biological pathways were analyzed for gene set enrichment in the differentiated (DIFF) phenotype. (A) A butterfly plot was generated to show the positive and negative associations of genes based on their rank and ranking metric score in the GSEA, providing a clear view of how gene expression changes correlate with HC11 differentiation. (B) Representative enrichment plots for significantly enriched pathways, including mTOR, prolactin, insulin, autophagy, and ErbB-associated gene sets. (C) KEGG gene–pathway interaction network constructed using Cytoscape, displaying genes (oval nodes) and pathways (hexagonal nodes) with shared interactions. Pathways are color coded as positively enriched (red) or negatively enriched (green), while genes are shown as up-regulated (orange) or down-regulated (blue) based on fold change (FC) ≥ 2. This network consists of 138 nodes and 336 edges, illustrating the complex interactions between pathways and genes.

Among 56 KEGG pathways evaluated, 15 exhibited positive enrichment in the DIFF phenotype, whereas 41 showed negative enrichment (Table 1). Of the positively enriched gene sets, 5 pathways, namely, mTOR, prolactin, insulin, autophagy, and ErbB signaling, were statistically significant. Conversely, 36 of the negatively enriched pathways were statistically significant and included gene sets associated with p53, Wnt, FoxO, PI3K–Akt, EGFR, Rap1, HIF-1, JAK–STAT, and apoptosis signaling pathways. A butterfly plot (Fig. 5A) displays the distribution and ranking metric of positively and negatively enriched gene sets across the ranked expression profile, while enrichment plots (Fig. 5B) illustrate the top 5 positively enriched pathways. Representative normalized enrichment plots for negatively enriched KEGG pathways are provided in Fig. S2. The KEGG gene–pathway interaction network constructed using Cytoscape (Fig. 5C) revealed 138 nodes and 336 edges, with most genes shared by multiple pathways, indicating extensive cross talk between signaling mechanisms during differentiation.

**Table 1.** Gene set enrichment analysis (GSEA) of KEGG biological pathways dysregulated during differentiation *

Sr	Pathway	Size	ES	NES	NOM *P* value	FDR *q* value	FWER *P* value	Rank at max	Sig.
Up-regulated pathways during differentiation based on whole-gene expression									
**1**	**mTOR**	**140**	**0.29**	**1.57**	**0.000**	**0.127**	**0.057**	**1,377**	**Yes**
**2**	**Prolactin**	**76**	**0.42**	**1.54**	**0.000**	**0.100**	**0.112**	**989**	**Yes**
**3**	**Insulin**	**130**	**0.35**	**1.40**	**0.000**	**0.130**	**0.258**	**1,794**	**Yes**
**4**	**Autophagy**	**137**	**0.27**	**1.34**	**0.000**	**0.146**	**0.303**	**1,677**	**Yes**
**5**	**ErbB**	**85**	**0.26**	**1.33**	**0.000**	**0.143**	**0.303**	**1,378**	**Yes**
6	VEGF	74	0.25	1.19	0.227	0.293	0.670	2,469	NS
7	AMPK	88	0.25	1.18	0.000	0.290	0.670	1,618	NS
8	Estrogen	119	0.25	1.12	0.096	0.341	0.760	2,996	NS
9	PPAR	70	0.36	1.10	0.268	0.324	0.760	3,395	NS
10	Tumor formation	79	0.34	1.10	0.295	0.304	0.760	1,360	NS
11	Glutathione	46	0.26	1.06	0.200	0.335	0.820	1,697	NS
12	NF-κB	97	0.22	0.83	0.597	0.780	1.000	1,624	NS
13	Transcription factor basal	39	0.24	1.10	0.000	0.416	1.000	12,563	NS
14	Endocytosis	258	0.14	1.01	0.667	0.529	1.000	3,640	NS
15	Glycolysis–gluconeogenesis	59	0.42	1.05	0.333	0.500	1.000	3,131	NS
Down-regulated pathways during differentiation based on whole-gene expression									
**1**	**Viral carcinogenesis**	**180**	**−0.30**	**−1.55**	**0.000**	**0.014**	**0.100**	**3,320**	**Yes**
**2**	**Transcription cancer**	**158**	**−0.34**	**−1.31**	**0.000**	**0.041**	**0.400**	**3,071**	**Yes**
**3**	**Epigenetic regulators**	**35**	**−0.59**	**−1.70**	**0.000**	**0.055**	**0.000**	**1,369**	**Yes**
**4**	**p53 signaling**	**70**	**−0.42**	**−1.64**	**0.000**	**0.055**	**0.055**	**3,197**	**Yes**
**5**	**Hedgehog signaling**	**79**	**−0.46**	**−1.61**	**0.000**	**0.055**	**0.055**	**2,570**	**Yes**
**6**	**Wnt signaling**	**140**	**−0.39**	**−1.60**	**0.000**	**0.055**	**0.055**	**3,679**	**Yes**
**7**	**Β cell receptor**	**75**	**−0.37**	**−1.58**	**0.000**	**0.055**	**0.055**	**2,343**	**Yes**
**8**	**Transcription**	**157**	**−0.43**	**−1.52**	**0.000**	**0.115**	**0.112**	**1,807**	**Yes**
**9**	**Hippo signaling**	**148**	**−0.31**	**−1.48**	**0.000**	**0.115**	**0.156**	**3,679**	**Yes**
**10**	**FoxO signaling**	**93**	**−0.35**	**−1.45**	**0.000**	**0.108**	**0.156**	**2,699**	**Yes**
**11**	**Transcription factors**	**86**	**−0.45**	**−1.45**	**0.000**	**0.102**	**0.156**	**2,530**	**Yes**
**12**	**PI3–AKT**	**310**	**−0.32**	**−1.37**	**0.000**	**0.137**	**0.289**	**3,526**	**Yes**
**13**	**Breast cancer**	**139**	**−0.34**	**−1.37**	**0.000**	**0.135**	**0.289**	**3,305**	**Yes**
**14**	**Breast–mammary**	**139**	**−0.34**	**−1.37**	**0.000**	**0.123**	**0.289**	**3,305**	**Yes**
**15**	**Immune response**	**95**	**−0.40**	**−1.37**	**0.000**	**0.135**	**0.322**	**2,051**	**Yes**
**16**	**Type II interferon**	**65**	**−0.45**	**−1.36**	**0.000**	**0.124**	**0.322**	**2,051**	**Yes**
**17**	**Inflammation**	**46**	**−0.53**	**−1.35**	**0.000**	**0.120**	**0.322**	**1,873**	**Yes**
**18**	**Cell proliferation**	**155**	**−0.40**	**−1.34**	**0.000**	**0.119**	**0.322**	**1,111**	**Yes**
**19**	**Metabolism**	**130**	**−0.43**	**−1.33**	**0.000**	**0.120**	**0.322**	**1,882**	**Yes**
**20**	**Cancer mouse**	**511**	**−0.34**	**−1.31**	**0.000**	**0.134**	**0.369**	**4,411**	**Yes**
**21**	**Calcium signaling**	**219**	**−0.35**	**−1.31**	**0.000**	**0.130**	**0.369**	**3,972**	**Yes**
**22**	**PI3–AKT–mTOR**	**395**	**−0.29**	**−1.30**	**0.000**	**0.129**	**0.369**	**3,364**	**Yes**
**23**	**EGFR signaling**	**38**	**−0.43**	**−1.28**	**0.000**	**0.138**	**0.369**	**1,234**	**Yes**
**24**	**Rap1 signaling**	**205**	**−0.30**	**−1.28**	**0.000**	**0.135**	**0.369**	**2,870**	**Yes**
**25**	**HIF-1 signaling**	**68**	**−0.22**	**−1.27**	**0.000**	**0.144**	**0.422**	**2,075**	**Yes**
**26**	**Notch signaling**	**57**	**−0.49**	**−1.25**	**0.000**	**0.165**	**0.539**	**4,790**	**Yes**
**27**	**TGF-β signaling**	**102**	**−0.36**	**−1.24**	**0.000**	**0.171**	**0.539**	**3,060**	**Yes**
**28**	**T cells**	**106**	**−0.29**	**−1.24**	**0.092**	**0.168**	**0.539**	**3,976**	**Yes**
**29**	**TNF signaling**	**110**	**−0.34**	**−1.23**	**0.166**	**0.166**	**0.539**	**3,302**	**Yes**
**30**	**Cytokines**	**201**	**−0.37**	**−1.21**	**0.092**	**0.174**	**0.583**	**3,019**	**Yes**
**31**	**JAK–STAT**	**147**	**−0.30**	**−1.20**	**0.176**	**0.180**	**0.583**	**3,577**	**Yes**
**32**	**Cell cycle**	**121**	**−0.45**	**−1.19**	**0.000**	**0.185**	**0.583**	**4,382**	**Yes**
**33**	**Apoptosis**	**142**	**−0.21**	**−1.17**	**0.075**	**0.211**	**0.699**	**4,618**	**Yes**
**34**	**ECM**	**84**	**−0.40**	**−1.16**	**0.196**	**0.212**	**0.699**	**4,171**	**Yes**
**35**	**Focal adhesion**	**195**	**−0.27**	**−1.15**	**0.202**	**0.224**	**0.749**	**4,211**	**Yes**
**36**	**Chemokines**	**166**	**−0.25**	**−1.12**	**0.271**	**0.247**	**0.749**	**3,548**	**Yes**
37	RAS signaling	221	−0.25	−1.11	0.170	0.252	0.749	3,473	NS
38	MAPK signaling	300	−0.24	−1.07	0.254	0.287	0.802	3,976	NS
39	cAMP signaling	190	−0.26	−1.05	0.366	0.314	0.846	3,934	NS
40	Phosphatidylinositol	82	−0.20	−1.18	0.143	0.112	0.900	3,553	NS
41	Viral protein interaction	74	−0.35	−1.11	0.500	0.263	1.000	2,584	NS

* Pathways in bold are significant.

KEGG, Kyoto Encyclopedia of Genes and Genomes; ES, enrichment score; NES, normalized enrichment score; FDR, NOM *P* value, normalized *P* value; false discovery rate; FWER, family-wise error rate; mTOR, mechanistic target of rapamycin; ErbB, erythroblastic oncogene B; VEGF, vascular endothelial growth factor; AMPK, AMP-activated protein kinase; PPAR, peroxisome proliferator-activated receptor; NF-κB, nuclear factor kappa-light-chain-enhancer of activated B cells; p53, tumor protein 53 (TP53); FoxO, forkhead box O transcription factor; PI3K–AKT, phosphoinositide 3-kinase–protein kinase B; EGFR, epidermal growth factor receptor; Rap1, Ras-related protein 1; HIF-1, hypoxia-inducible factor 1; TGF-β, transforming growth factor beta; TNF, tumor necrosis factor; JAK–STAT, Janus kinase–signal transducer and activator of transcription; ECM, extracellular matrix; RAS, rat sarcoma; MAPK, mitogen-activated protein kinase; cAMP, cyclic adenosine monophosphate; NS, nonsignificant

### Regulation of TFs and ERs in HC11 differentiated cells

Transcription machinery is integral to cell fate and differentiation, while ERs provide the necessary gene expression stability to improve clarity and readability [41,42]. To characterize the regulatory architecture underlying the differentiation condition, we analyzed DEGs with FC > 1 and identified a total of 172 (57 up- and 115 down-regulated) TFs and 69 (14 up- and 55 down-regulated) ERs (Table S7). Among the up-regulated TFs, several were directly associated with lineage commitment and cell fate determination, including Zbtb16, FoxO1, Sox13, Dlx5, Id1/3, and Sohlh1. Other TFs, such as Srebf1, Klf6, Klf15, and Trib3, were linked to metabolic reprogramming. Differential expression of TFs like Glis3, Arntl, Grin1, and Lmo1 suggests roles in neuroendocrine and circadian regulation, while Atf3, Atf6, Ddit3, and Nupr1 indicate activation of stress-adaptive transcriptional responses. Dual-role regulators, such as Myc, Sp100, Taf4b, and Zfpm1, were also observed. The down-regulated TFs included those related to proliferation (E2f8, Ccnd1, and Mki67), developmental signaling (Wnt5a, Notch3, and Shox2), immune response (Irf5, Il1rl1, and Cd7), and alternative lineage specification (Sox5, Sox11, and Zeb1).

Among the 14 up-regulated ERs, key factors included FoxO1, Gadd45a, Gadd45g, Cited1, and Hist1h1e, all associated with chromatin remodeling, DNA demethylation, and transcription. Other regulators like Sp100, Zfpm1, Actr3b, Arrb1, and Arid5a contribute to chromatin restructuring, histone modifications, and immune-related transcriptional control. The 55 down-regulated ERs included chromatin remodelers (Smarca1 and Hmgn2), histone modifiers (Hdac9, Padi4, and Phf21b), DNA methylation regulators (Uhrf1), and genome stability mediators (Rad51, Brca1, and Aurkb). Notably, 38 genes were identified as dual-function regulators, classified as both TFs and ERs, including Myc, FoxO1, Bard1, Sox5, Sox11, Prdm1, Arid5a, and Zbtb7c.

### Data comparison with a previously published study of Sornapudi et al.

**Fig. 6.**
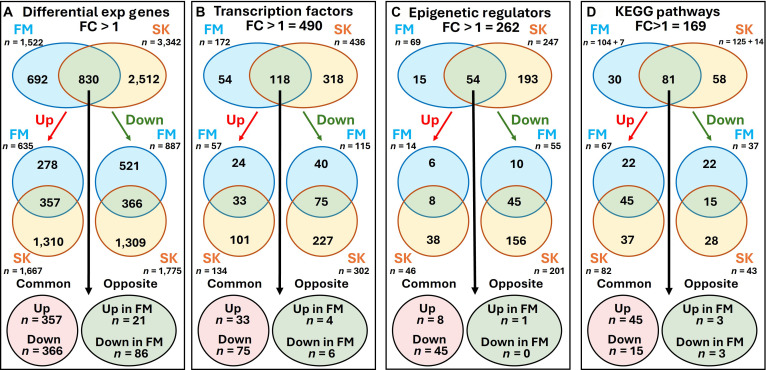
Comparative analysis of differentially expressed genes (DEGs), transcription factors, epigenetic regulators, and Kyoto Encyclopedia of Genes and Genomes (KEGG) pathways in our (FM) and Sornapudi et al. (SK) studies. Venn diagrams illustrate the overlap between FM (blue) and SK (yellow) groups for (from left to right): (A) DEGs, (B) transcription factors, (C) epigenetic regulators, and (D) enriched KEGG pathways with fold change (FC) > ± 1. Each main category is further subdivided into up-regulated (red arrows) and down-regulated (green arrows) gene sets. The lower panels show the number of overlapping genes/pathways that are specifically up-regulated or down-regulated in the FM group. The intersecting numbers represent shared elements, while the nonoverlapping areas indicate unique features of each group. These results highlight both the common and distinct regulatory mechanisms observed in FM and SK studies.

Previously, Sornapudi et al. (SK) [9,10] reported transcriptomic changes during HC11 lactogenic differentiation, with particular emphasis on transcriptional and epigenetic regulatory networks. To evaluate concordance and divergence between studies, we performed a comparative analysis of DEGs, TFs, ERs, and enriched KEGG pathways between our dataset (FM) and the SK dataset. Comparative analyses were conducted using the published DEG and pathway lists reported by SK rather than reprocessing their raw sequencing data. Accordingly, the SK dataset was not renormalized or reanalyzed within our analytical pipeline, and observed differences may therefore reflect both biological variation and differences in normalization strategies, statistical models, and DEG selection criteria between studies. To improve comparability, FC thresholds were aligned (FC ≥ 1) for interstudy analyses where appropriate. A detailed comparison is provided in Table S7. The Venn diagrams in Fig. 6 provide a visual summary of these comparisons. In total, 1,522 DEGs (FC > ±1) were identified in the FM group, compared to 3,342 DEGs in the SK group, with 830 genes commonly dysregulated between both (Fig. 6A). To determine whether this overlap exceeded random expectation, a hypergeometric test was performed using the 15,582 shared detectable genes as background. The observed overlap was highly significant (*P* value < 2.2 × 10^−16^), indicating strong concordance between the 2 datasets. Specifically, our study identified 635 (41.7%) up-regulated and 887 (58.3%) down-regulated genes, whereas the SK group reported 1,667 (49.9%) up-regulated and 1,775 (50.1) down-regulated genes. Notably, the expression of a high number of genes (357 up-regulated and 366 down-regulated genes) matched across both datasets, suggesting a core set of genes consistently modulated during differentiation (Fig. 6A). Only a small subset of genes (*n* = 107) showed discordant expression; i.e., 21 genes that were down-regulated in the SK group appeared up-regulated in FM, while 86 genes that were up-regulated in SK were found down-regulated in our dataset (Fig. 6A). Directional concordance was formally evaluated using Fisher’s exact test. Concordant regulation significantly exceeded discordant cases (odds ratio [OR] = 72.3; *P* value < 2.2 × 10^−16^), confirming that shared DEGs are directionally consistent beyond random expectation.

Out of 490 TFs showing FC > ±1, 118 were shared between FM and SK, while 54 and 318 were uniquely altered in FM and SK, respectively (Fig. 6B). Among the shared TFs, 33 were commonly up-regulated and 75 were commonly down-regulated, whereas 21 exhibited inverse expression trends. Fisher’s exact testing demonstrated significant directional agreement among shared TFs (OR ≈ 27.5; *P* value < 1 × 10^−12^), supporting conservation of transcriptional regulatory architecture across studies. Similarly, 54 ERs were commonly affected in both groups, with 15 and 193 uniquely altered in FM and SK, respectively (Fig. 6C). Of the shared regulators, 8 were commonly up-regulated and 45 were commonly down-regulated, with only 1 discordant case. Directional concordance among shared ERs was also statistically significant (*P* value < 1 × 10^−10^), further reinforcing regulatory overlap.

To evaluate potential functional similarities and differences between the FM and SK groups, we performed KEGG pathway enrichment analysis using genes with FC > ±1 (*n* = 169). In our dataset, 104 KEGG pathways were associated with either up- or down-regulated genes, and an additional 7 pathways appeared in both up and down categories. In the SK group, 125 pathways were associated with either up- or down-regulated genes, and 14 were common to both up and down groups (Fig. 6D). After removing pathways appearing in both up- and down-regulated categories within each study, 81 nonredundant pathways were shared between datasets, with 30 and 58 uniquely enriched in FM and SK, respectively. Fisher’s exact test demonstrated that shared pathway enrichment significantly exceeded random expectation (OR = 2.6; *P* value = 0.002), supporting functional concordance between studies. Of the shared pathways, 45 were consistently up-regulated and 15 were consistently down-regulated, while only 6 showed opposing trends (see Table S7 for details). Fisher’s exact testing confirmed significant directional concordance of pathway regulation between studies (OR ≈ 75; *P* value < 1 × 10^−10^). Collectively, these quantitative analyses demonstrate that the overlap between the FM and SK datasets is not only statistically significant but also directionally concordant, supporting the presence of a conserved transcriptional program underlying HC11 lactogenic differentiation despite differences in experimental design.

### Condition-restricted gene detection during HC11 differentiation

To better understand the transcriptional dynamics associated with mammary epithelial cell differentiation, we identified subsets of genes exhibiting condition-restricted detection patterns (i.e., uniquely expressed). Using a defined threshold of TPM > 1 in both biological replicates of one condition and TPM = 0 in both replicates of the other condition, we identified 19 genes with FC > ±2 that were detected above the threshold exclusively in differentiated HC11 cells. These genes included Csn1s2a, Csn2, Esp31, Esp34, Glod5, Igfn1, Klk1b16, Klk1b21, Klk1b22, Klk1b27, Klk1b4, Klk1b5, Spink8, Sult1d1, Sval2, Tfr2, Timp4, Wap, and Zbtb16. Conversely, 9 genes (Csf2rb, Gdf2, Gm21466 [Spin2i], Gm3757 [Spin2-ps9], Hao2, Insl3, Lrrc15, Mrgprb8, and Pcdhgb5) were detected above the threshold in undifferentiated cells but were below the detection threshold in differentiated samples.

**Fig. 7.**
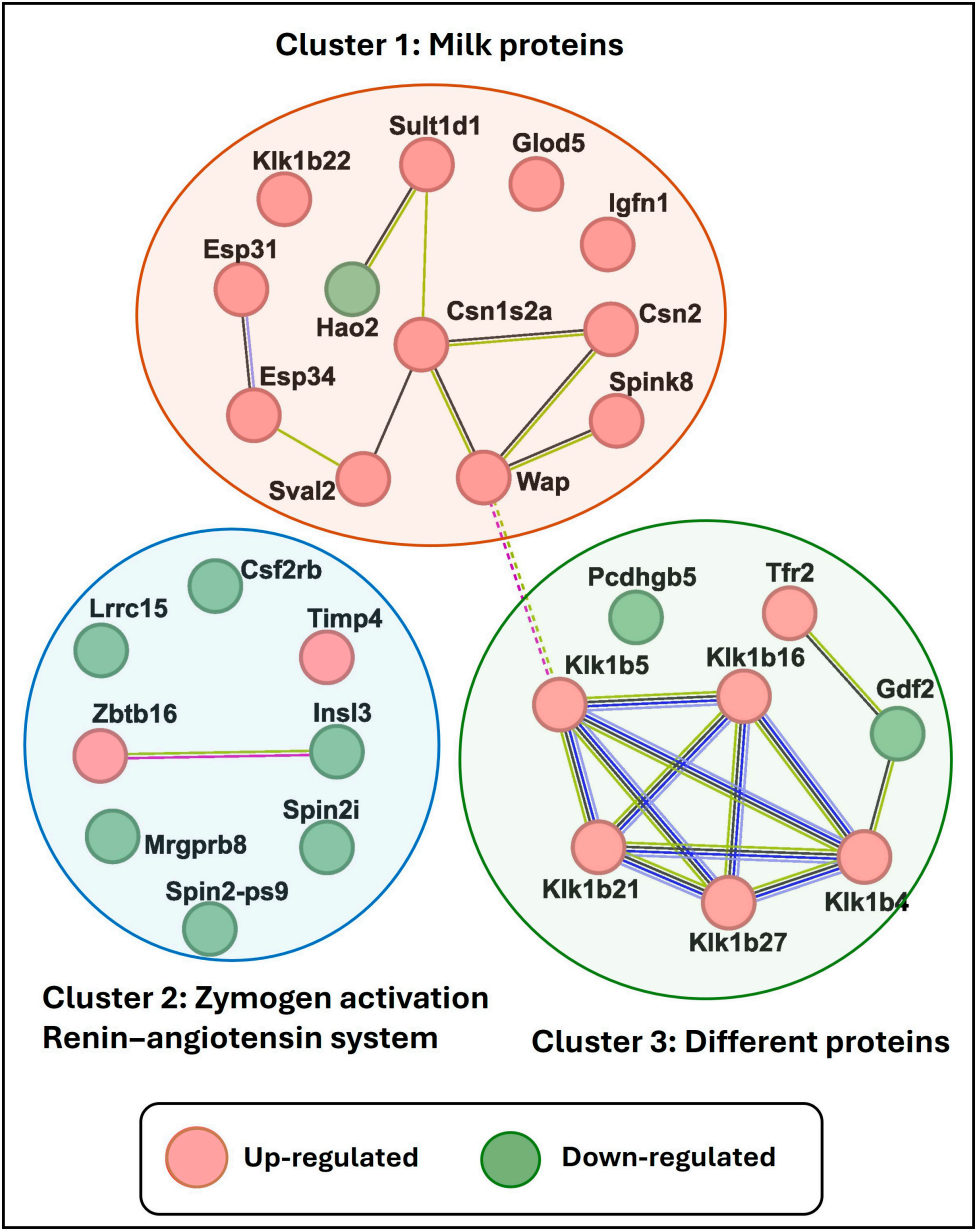
Clustering of unique genes regulated during mammary epithelial cell differentiation using STRING. Genes are grouped into 3 functional clusters based on pathway and biological role enrichment: Cluster 1 represents genes involved in milk protein production, cluster 2 includes genes associated with zymogen activation and the renin–angiotensin system, and cluster 3 contains genes encoding miscellaneous or distinct protein classes. Nodes represent individual genes with fold change (FC) > 2, with red nodes indicating only those appearing during differentiation and green nodes indicating genes expressed during undifferentiated conditions. Edges between genes denote known or predicted interactions, such as coexpression or shared pathways.

Complete TPM values for these 28 genes are provided in Table S8. Importantly, classification as condition restricted reflects the applied detection threshold rather than confirmed biological absence. Given the limited number of biological replicates and the inherent variability of low-abundance transcripts, these genes should be interpreted as being below the limit of reliable detection rather than absent. Accordingly, these observations are descriptive of transcript detection patterns and should be considered cautiously without inferring definitive condition-specific gene expression. During mammary epithelial cell differentiation, the activation and suppression of specific gene sets are essential to support the transition from a proliferative to a functionally specialized, milk-producing state. Based on these uniquely expressed gene sets, we created gene networks, visualized in Fig. 7 using STRING, that formed 3 major clusters. To no surprise, cluster 1 was dominated by milk protein genes, including Csn1s2a, Csn2, Wap, and Sult1d1, all of which were strongly up-regulated during differentiation. These genes are crucial for establishing and maintaining lactation-specific functions. Notably, the up-regulation of Csn1s2a, Csn2, and Wap in cluster 1 underscores the commitment of differentiated epithelial cells to milk production, as these genes encode caseins and whey acidic protein, core components of milk essential for neonatal nutrition [43–45]. The concurrent expression of Sult1d1, Glod5, and Spink8 suggests the induction of metabolic and protease inhibitory mechanisms that may protect milk integrity and regulate secretion [46–48]. Similarly, cluster 2 encompassed genes such as Klk1b5, Klk1b16, Klk1b21, and Klk1b27, primarily associated with zymogen activation and the renin–angiotensin system, suggesting a coordinated up-regulation of proteolytic processes potentially contributing to extracellular matrix remodeling, tissue restructuring, and paracrine signaling required for full differentiation. Interestingly, this cluster also includes a few down-regulated genes (Gdf2, Pcdhgb5, and Tfr2), which may play inhibitory or regulatory roles during differentiation. The presence of these down-regulated factors in this cluster may indicate a suppression of pathways incompatible with terminal differentiation.

Cluster 3 groups a variety of distinct functional proteins, including TFs (Zbtb16), cytokine receptors (Csf2rb), and hormonal genes (Insl3), suggesting that the repression or fine-tuning of broader developmental pathways is necessary to reinforce lineage commitment. Overall, this network analysis reveals that mammary differentiation involves a coordinated regulation of genes responsible for lactation, protease activity, and broader cellular functions. These distinct gene clusters reflect the functional specialization required for the differentiated epithelial phenotype.

## Discussion

This study presents a systems-level view of HC11 mammary epithelial cell differentiation using RNAseq. Bulk RNAseq profiling revealed clear transcriptional separation between undifferentiated and differentiated HC11 cells and identified 566 high-confidence DEGs despite population heterogeneity. These data support a robust differentiation-associated transcriptional program for downstream functional interpretation. Importantly, the global distribution of transcript abundance remained comparable between conditions, suggesting that differentiation induces targeted gene regulatory remodeling rather than widespread transcriptome destabilization. Together, these findings establish a statistically robust framework for interpreting pathway enrichment, network topology, and regulatory integration underlying HC11 differentiation.

In our differentiated HC11 mammary epithelial cells, we observed strong up-regulation of terminal differentiation markers, including Wap, Csn2, Lpl, and Cd36, reflecting functional maturation and commitment to a secretory epithelial phenotype. Additionally, key markers of lactogenic differentiation, including Lalba, Btn1a1, Xdh, Gata3, and C/EBPβ (Cebpb), also showed elevated expression, further confirming the establishment of a differentiated transcriptional profile. Interestingly, Nr3c1 (glucocorticoid receptor) and Elf5, which are typically associated with early stages of hormonal priming and lineage commitment, exhibited reduced transcript levels in differentiated cells [49]. This may reflect their transient role during the early differentiation window, followed by down-regulation once cells achieve phenotypic stabilization. Although Stat5 and Akt1 mRNA expression increased only modestly and without statistical significance, its downstream targets, Wap and Csn2, were markedly up-regulated, suggesting that STAT5 activity may be regulated posttranscriptionally via phosphorylation rather than through changes in mRNA abundance. Notably, in our study, apoptosis-related signaling pathways like NF-κB, TNF, p53, Hif-1, Hippo and MAPK, and cell cycle [50,51] were significantly down-regulated, which aligns with the emerging understanding that active Stat5 not only promotes differentiation but also supports cell survival by repressing pro-apoptotic signaling and enhancing self-sufficiency in growth signals [52]. This dual role of Stat5 ensures both functional maturation and longevity of differentiated mammary epithelial cells. Interestingly, Prlr expression remained unchanged (low), consistent with previous reports indicating that receptor levels may not vary significantly during the later stages of HC11 differentiation, yet signaling responsiveness may still be robust [10,53,54]. Together, these findings illustrate the layered regulation of mammary differentiation, where transcriptional, posttranscriptional, and signaling dynamics converge to enable functional specialization. Thus, further exploration of protein-level changes and functional validation (e.g., phospho-STAT5 and PRLR signaling) would provide a more complete understanding of differentiation status in this model.

To elucidate the molecular framework underlying mammary epithelial cell differentiation, we performed comprehensive functional and pathway enrichment analyses on DEGs identified through the RNAseq profiling of undifferentiated and differentiated HC11 cells. GO profiling revealed marked transcriptional shifts across biological processes and molecular functions, with significant enrichment in transmembrane transport, lipid metabolism, proteolysis, and signal transduction, key pathways supporting secretory maturation. The predominance of plasma membrane and extracellular-associated genes reflects surface remodeling critical for cellular environment communication. Up-regulated cytokine activity, ion binding, and enzymatic functions further supported structural reorganization and the acquisition of secretory competency during lactogenic differentiation.

Functional biological pathway analysis, integrating GO terms, PPI networks and KEGG pathways, pinpointed several biological pathways and interconnected genes as central regulators of the differentiation process. These pathways not only govern metabolic activity and cellular growth but also intersect to form a tightly regulated signaling axis necessary for the switch from proliferation to functionally mature states. The overlap of DEGs across multiple signaling routes further highlights the pleiotropic roles of key genes in orchestrating this transition. Network topology analysis identified 10 genes (Agt, Ccnd1, Igf1, Mki67, Myc, Calm4, Rasgrap1, Cd69, Il6, and Pecam1) as highly connected nodes within the differentiation-associated PPI network, using the maximum clique centrality algorithm implemented in CytoHubba. It is important to emphasize that hub status reflects network centrality rather than direct causal dominance.

Several of the identified hub genes have established roles in mammary gland biology, supporting their biological plausibility. For example, Ccnd1 and Myc are key regulators of cell-cycle progression and mammary epithelial proliferation, and their modulation is tightly linked to alveolar development and differentiation [55,56]. Igf1 signaling has been shown to cooperate with prolactin and insulin pathways in regulating mammary epithelial growth and lactogenic differentiation [57,58]. Likewise, Il6 participates in mammary gland remodeling and inflammatory signaling associated with developmental transitions [59,60]. Mki67, a canonical proliferation marker, reflects the well-characterized exit from the proliferative state during terminal differentiation [61].

In contrast, other identified hubs, such as Calm4, Rasgrap1, Cd69, and Pecam1, are less extensively characterized in mammary epithelial differentiation. Notably, Cd69 and Pecam1 are traditionally associated with immune or endothelial contexts; however, their appearance as network-central nodes may reflect transient cytokine signaling, stress-response pathways, or epithelial–microenvironment communication during differentiation rather than direct involvement in lineage conversion. Their presence may also arise from shared pathway membership within highly interconnected signaling modules, highlighting the potential influence of network topology on hub ranking. Overall, hub gene analysis provides a useful prioritization framework, but these candidates should be interpreted as hypothesis generating rather than definitive mechanistic drivers, with functional validation required to establish their roles in mammary epithelial differentiation.

**Fig. 8.**
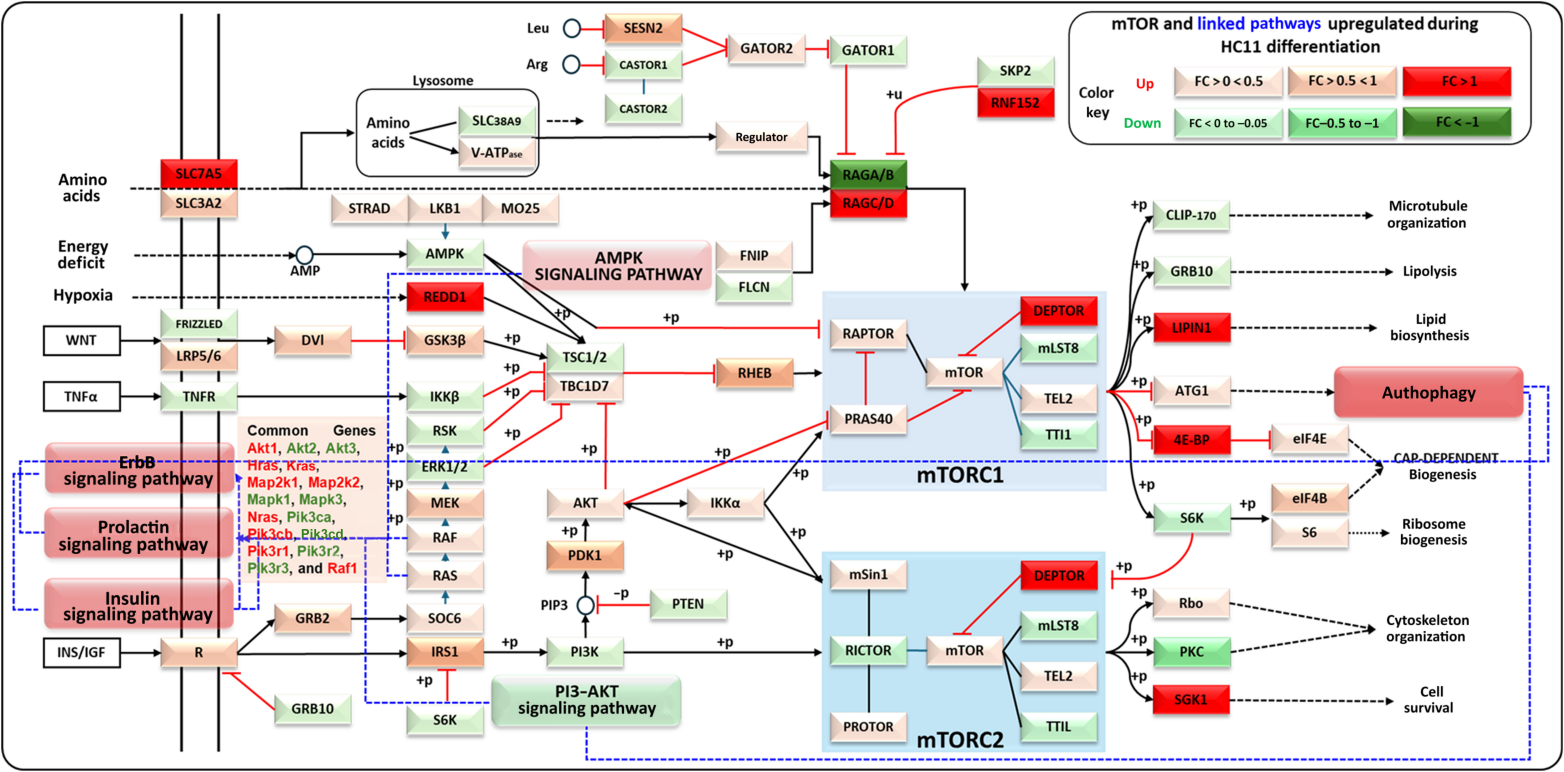
Network of significantly up-regulated pathways during HC11 differentiation with mTOR as the central hub. The figure illustrates the interconnections among the 5 statistically significant up-regulated pathways: mTOR, prolactin, insulin, autophagy, and ErbB signaling. The mTOR pathway is highlighted as the central node due to its key role in integrating signals related to nutrient sensing, protein synthesis, and cellular growth. Genes identified as transcriptionally altered in this study are highlighted and color coded (red: up-regulated; green: down-regulated), whereas additional pathway components are shown to provide biological context (red: positively enriched; green: negatively enriched). mTORC1 and mTORC2 (blue boxes) receive inputs from PI3K–AKT, RHEB, and upstream nutrient/energy sensors (AMPK/REDD1). Prolactin, insulin, and ErbB pathways feed into PI3K–AKT and STAT5 signaling, while autophagy nodes interface with mTORC1 via ULK1/ATG regulation. Arrows and connections represent functional relationships and interactions between these pathways, emphasizing their collective contribution to cellular differentiation processes. +p/−p, after phosphorylation/dephosphorylation; +u, after ubiquitinoylation; dashed black arrows, indirect connection; solid black arrows, direct connection; dashed blue lines, pathways linked to mTOR during differentiation.

GSEA revealed coordinated transcriptional remodeling accompanying the transition from a proliferative to a lactogenic phenotype in HC11 cells (Fig. 8). Among the positively enriched gene sets, mTOR-associated networks emerged as a central integrative node linking hormonal, metabolic, and growth-factor-related transcriptional programs. To provide a structured overview of this interconnected signaling architecture, we constructed an integrative schematic summarizing transcriptionally enriched pathways and their interrelationships, with mTOR positioned as a central node (Fig. 8). Rather than functioning independently, prolactin, insulin, ErbB, and autophagy-associated gene sets display coordinated transcriptional alignment within this framework. Importantly, these observations reflect transcriptional associations rather than direct functional pathway activation and, therefore, should be interpreted as hypothesis generating. Definitive assessment of pathway activity and downstream functional consequences would require orthogonal validation at the protein and functional levels. Insulin signaling through the insulin receptor is known to engage the PI3K–AKT–mTOR axis and regulate alveologenesis, lipid biosynthesis, and translational capacity required for milk production [62–65]. Consistent with this framework, we observed increased transcript abundance of Pik3ca/Pik3cb, Akt1, Rheb, and Rps6kb1 in differentiated HC11 cells, reflecting coordinated enrichment of mTOR-related gene sets (Fig. 8) [62–67]. Notably, although the overall PI3K–AKT KEGG gene set displayed negative enrichment in GSEA, this does not necessarily contradict the observed up-regulation of selected axis components. GSEA evaluates coordinated regulation across the full pathway gene set, which includes multiple proliferation-associated and context-dependent regulators that were transcriptionally reduced during differentiation. Therefore, negative enrichment of the broader PI3K–AKT pathway likely reflects suppression of proliferative modules rather than uniform down-regulation of all signaling elements within the axis. The prolactin–STAT5 pathway, a principal hormonal regulator of lactogenic gene expression, promotes transcription of milk-associated genes such as Csn2 and Wap, which were also up-regulated in our dataset [2,68]. These prolactin-driven transcriptional programs increase biosynthetic demand, which is mechanistically linked to mTOR-regulated translational control in mammary epithelial cells.

ErbB signaling further interfaces with this regulatory network (Fig. 8). ErbB4 has been reported to cooperate with prolactin signaling through modulation of STAT5 activity, whereas EGFR (ErbB1) signaling is more commonly associated with proliferative states and is attenuated during differentiation [69,70]. The observed enrichment of ErbB-associated transcripts therefore supports coordinated reorganization of growth factor signaling consistent with differentiation-associated remodeling at the mTOR axis [62–67].

mTORC1 is widely recognized as a metabolic and biosynthetic hub. While mTORC1 activity is classically associated with phosphorylation of S6K1 and 4E-BP1 to enhance translation initiation and modulate autophagy, our findings reflect coordinated transcriptional enrichment of mTOR-related and autophagy-associated gene sets rather than direct biochemical measurement of pathway activation. Notably, selective autophagic processes, including mitophagy, remain engaged to support organelle QC during metabolic remodeling [71–74]. Up-regulation of mitophagy-related genes, such as Prkn (Parkin) and Deptor, further suggests balanced transcriptional regulation of anabolic and catabolic processes within mTOR-associated networks [75,76].

Taken together, these data support a model in which prolactin–STAT5, insulin–PI3K–AKT, and ErbB signaling converge transcriptionally on mTOR-centered pathways to facilitate the transition from a proliferative to a metabolically specialized, milk-producing epithelial phenotype. Importantly, this interpretation is consistent with prior functional studies demonstrating that pharmacological inhibition of mTORC1 (e.g., rapamycin or everolimus treatment) suppresses mammary epithelial differentiation and milk protein synthesis, as well as differentiation programs in other cellular systems. These published findings support the functional relevance of mTOR-associated networks while underscoring that the present study assesses transcriptional coordination rather than direct biochemical pathway activation.

With respect to autophagy, it is important to distinguish transcriptional enrichment of autophagy-related genes from functional autophagic flux. Although mTORC1 activation is classically associated with suppression of bulk autophagy, the observed enrichment of autophagy- and mitophagy-associated transcripts in our dataset may reflect selective or context-dependent regulatory adaptation rather than increased global autophagic activity. Notably, Deptor, an endogenous inhibitor of mTOR, was up-regulated, suggesting potential buffering or feedback regulation within the mTOR network rather than straightforward pathway activation. Similarly, increased expression of Prkn (Parkin) and other mitophagy-associated genes may indicate transcriptional priming of QC mechanisms during metabolic remodeling, rather than direct evidence of enhanced autophagic flux. Therefore, while our findings support coordinated transcriptional remodeling of mTOR- and autophagy-associated gene sets, definitive conclusions regarding bulk or selective autophagy activity would require dedicated flux assays or protein-level validation.

Importantly, GSEA reflects coordinated transcriptional changes within predefined pathways and does not directly measure functional pathway activity. For example, reduced expression of genes within the p53-associated gene set does not necessarily indicate diminished p53 signaling, as transcriptional repression of specific targets may occur in the context of complex regulatory feedback. Accordingly, our conclusions are framed in terms of transcriptional coordination rather than biochemical activation. Alongside, gene sets associated with proliferation, developmental plasticity, and stress signaling, including Wnt, Hedgehog, Notch, cell-cycle regulators, and inflammatory pathways, were transcriptionally reduced. Suppression of these programs is consistent with cell-cycle exit and stabilization of differentiated epithelial identity. Together, these coordinated transcriptional shifts illustrate how HC11 cells transition from a proliferative, stemlike state toward a metabolically specialized and functionally mature lactogenic phenotype [77–80].

Cellular differentiation is governed by coordinated changes in transcriptional networks and epigenetic architecture. In our study, transcriptomic profiling revealed extensive modulation of TFs and ERs, reflecting the complex reprogramming landscape underpinning HC11 cell differentiation. These factors were dynamically modulated during HC11 cell differentiation, reflecting a tightly controlled reprogramming landscape. Among 172 TFs, up-regulated subsets, including Zbtb16, FoxO1, Srebf1, and Sohlh1, were linked to lineage commitment, metabolic reprogramming, and stress adaptation, while down-regulated TFs, such as Ccnd1, Wnt5a, and Sox11, signaled repression of proliferative and developmental programs. Concurrently, 69 ERs showed coordinated changes: up-regulated ERs (e.g., Gadd45a, Cited1, and Hist1h1e) supported chromatin relaxation and lineage fidelity, whereas down-regulated ERs (e.g., Smarca1, Hdac9, and Uhrf1) reflected silencing of stemness-associated or proliferative chromatin states. Notably, 38 genes exhibited dual TF/ER roles, highlighting functional convergence between transcriptional control and epigenetic remodeling. This integrated regulatory shift underpins the establishment of a differentiated, biosynthetically active epithelial phenotype.

A previous transcriptomic study by SK [9] similarly examined HC11 differentiation, with particular emphasis on TF and epigenetic regulation during early lactogenic induction. Because raw SK sequencing data were not reprocessed within a unified analytical framework, differences between studies may reflect both biological and analytical variation. Although their analysis identified a larger number of DEGs using a FC threshold of ±1, a substantial conserved core of 830 genes was shared between both datasets, including consistent induction of canonical lactogenic markers such as Csn2, Wap, and Elf5. Hypergeometric testing confirmed that this overlap significantly exceeded random expectation, supporting the presence of a stable differentiation-associated transcriptional signature across independent studies. At the pathway level, both analyses revealed coordinated transcriptional remodeling consistent with transition from a proliferative to a specialized secretory state. Gene sets associated with insulin/IGF signaling, prolactin/STAT5 pathways, and mTOR-related networks were transcriptionally enriched, whereas pathways linked to cell-cycle progression, stress responses, and focal adhesion were reduced. Importantly, these findings reflect coordinated transcriptional shifts rather than direct biochemical pathway activation.

In addition to pathway-level concordance, network topology analysis further supported cross-study reproducibility. Of the 10 hub genes identified in our PPI network, 6 (Agt, Ccnd1, Il6, Mki67, Myc, and Pecam1) were also centrally positioned within the SK dataset, representing 60% overlap. This overlap was statistically significant and directionally concordant, indicating that key network-central nodes are conserved across studies. Several of these shared hubs, including Ccnd1, Myc, and Il6, have established roles in mammary epithelial proliferation and differentiation, reinforcing their biological plausibility. The remaining FM-specific hubs (Calm4, Rasgrp1, Cd69, and Igf1) likely reflect context-dependent network configuration rather than topological artifacts. While hub status reflects connectivity rather than causality, the convergence of regulatory centrality across independent datasets strengthens confidence in the core differentiation-associated network architecture. Overall, the concordance between gene-level overlap, pathway enrichment, and network-central nodes reinforces the reproducibility of the transcriptional program underlying HC11 lactogenic differentiation, while minor differences likely reflect variations in experimental design and differentiation staging rather than fundamental biological divergence.

Both studies reveal a broadly conserved transcriptional architecture underlying HC11 lactogenic differentiation. In agreement with SK, genes associated with proliferation and cell-cycle progression including Mki67, Foxm1, members of the E2f family, and chromatin regulators such as Uhrf1 and Chaf1b were consistently down-regulated, reflecting exit from the proliferative state. Conversely, TFs linked to differentiation, lipid metabolism, and metabolic adaptation, including FoxO1, Srebf1, Zbtb16, and Klf15, were enriched in differentiated cells. Together, these shared regulatory patterns support a conserved transition from a proliferative epithelial phenotype toward a specialized secretory state.

Despite this core concordance, differences were observed in the breadth and identity of TFs and ERs altered between datasets. A larger number of regulatory genes were uniquely modulated in the SK dataset, and a subset exhibited divergent expression trends. Such variability is not unexpected and likely reflects differences in experimental design, including hormone exposure regimens, serum composition, differentiation timing, and cell passage history. Even subtle variations in glucocorticoid type, induction duration, or culture conditions can influence chromatin accessibility, hormone responsiveness, and baseline transcriptional states in HC11 cells. Importantly, these differences do not undermine the conserved differentiation trajectory observed across studies. Rather, they highlight the context-dependent plasticity of mammary epithelial transcriptional networks and underscore how experimental parameters can shape the extent and timing of regulatory engagement. Overall, both analyses converge on a shared differentiation program characterized by suppression of proliferative circuits and coordinated transcriptional enrichment of metabolic and secretory pathways while allowing for variation in secondary regulatory modules.

In addition to confirming induction of canonical lactogenic markers and proteolytic components described by SK, our analysis identified additional differentiation-associated transcripts that refine the characterization of the lactogenic phenotype. For example, we observed selective enrichment of the Csn1s2a casein locus and coordinated regulation of multiple kallikrein family members (Klk1b16, Klk1b21, Klk1b22, and Klk1b27), suggesting structured modulation of proteolytic activity. Notably, the concurrent up-regulation of protease inhibitors (Spink8) and metabolic regulators (Sult1d1 and Glod5) points to balanced proteolytic control and redox adaptation accompanying milk protein synthesis. Rather than focusing solely on individual gene induction, we contextualized these transcriptional changes within coordinated signaling modules involving prolactin, insulin, and mTOR-associated networks. This integrative interpretation highlights how secretory activation is coupled to metabolic remodeling and protease regulation, providing a more comprehensive framework for understanding terminal HC11 differentiation. Together, our findings complement prior HC11 transcriptomic studies and contribute an expanded systems-level view of lactogenic transcriptional reprogramming.

### Limitations of the study

This study has several limitations that should be considered. RNAseq was performed using 2 independent biological replicates per condition. Although sequencing depth exceeded 100 million reads per sample and dispersion modeling indicated stable variance estimation, limited replication reduces statistical power and warrants cautious interpretation of differential expression results. The study was conducted in a single *in vitro* murine mammary epithelial cell line (HC11), which, while well established for lactogenic differentiation studies, does not fully recapitulate the complexity of *in vivo* mammary gland biology or human breast physiology. Additionally, bulk RNAseq was used; therefore, potential cellular heterogeneity within differentiated cultures cannot be resolved at a single-cell resolution. Pathway enrichment analyses reflect coordinated transcriptional changes but do not directly demonstrate biochemical activation, particularly for signaling pathways such as mTOR that are primarily regulated posttranslationally. Functional validation through protein-level assays or pharmacological perturbation (e.g., rapamycin treatment) was beyond the scope of this transcriptomic investigation. Despite these limitations, the reproducibility across replicates and concordance with previously published datasets support the robustness of the core differentiation-associated transcriptional program identified.

## Conclusions

In summary, our study provides an integrated, high-resolution transcriptomic landscape of HC11 mammary epithelial cell differentiation, capturing the multifaceted regulatory changes that drive the transition from a proliferative to a functionally mature, lactogenic state. Through a comprehensive RNAseq analysis, functional annotation, signaling network mapping, and comparative profiling, we identified distinct transcriptional signatures, pathway-associated transcriptional changes, and epigenetic shifts that support this process. Central signaling pathways, particularly mTOR, prolactin, insulin, and ErbB, emerged as convergent regulators orchestrating biosynthetic readiness and secretory specialization. The identification of uniquely expressed genes and hub regulators further highlights potential molecular markers and effectors of mammary gland maturation. Importantly, comparison with existing datasets underscores both conserved and divergent regulatory programs shaped by experimental context and temporal modulation. These findings not only deepen our understanding of lactogenic differentiation but also offer a valuable resource for dissecting lineage-specific gene regulation.

## Future Directions

Future studies should aim to explore dynamic temporal regulation, posttranscriptional mechanisms, and cell-specific functional assays to validate key effectors. Integration with *in vivo* models may further refine our understanding of lactogenic programming, offering translational insights into mammary gland development and lactation biology. The present transcriptomic analysis also generates several testable hypotheses. First, given the coordinated transcriptional enrichment of mTOR-associated networks and canonical lactogenic markers, we hypothesize that targeted disruption of upstream regulators such as Igf1 or Rheb would impair casein expression and attenuate differentiation-associated translational capacity. Second, the identification of conserved hub genes (Ccnd1, Myc, and Il6) across independent datasets suggests that modulation of these network-central nodes may alter the balance between proliferative exit and secretory commitment during HC11 differentiation. Third, enrichment of autophagy- and mitophagy-associated transcripts alongside mTOR-centered signaling raises the possibility that selective QC mechanisms are required to support metabolic remodeling during lactogenic transition. These hypotheses will require validation through functional perturbation studies, including assessment of autophagic flux and mTOR pathway activity.

## Ethical Approval

This is a cell-line-based study that did not involve any animals or humans; therefore, no ethical approval is required.

## Data Availability

The raw data generated during the current study can be downloaded from the NCB BioProject server, https://www.ncbi.nlm.nih.gov/bioproject/?term=PRJNA915407. In addition, the processed normalized count matrix and the complete DESeq2 statistical output (including log_2_ FCs, *P* values, and adjusted *P* values) are provided as supplementary data files in Excel format (Tables S1 and S2).
