# Supplementary material for: Shaping Cell Identity: Global Transcriptome and Pathway Shifts during Mouse Mammary Epithelial Cell Differentiation
Source: Comput Struct Biotechnol J. 2026 May 4;35(1):0055. doi: 10.34133/csbj.0055 (PMC13136624; doi:10.34133/csbj.0055)
Supplement: Supplementary 1 — Tables S1 to S8 Figs. S1 and S2 [file csbj.0055.f1.zip › Supplementary Data Figures.docx]

**Supplementary Data**

**Shaping Cell Identity: Global Transcriptome and Pathway Shifts During Mouse Mammary Epithelial Cell Differentiation**

Waqar Ahmad^1^*, Neena Gopinathan Panicker^1^*, Tahir A. Rizvi^2,3§^, and Farah Mustafa^1,3§^

^1^Department of Biochemistry and Molecular Biology,

^2^Department of Microbiology and Immunology,

College of Medicine & Health Sciences (CMHS), and

^3^Zayed Center for Health Sciences (ZCHS),

United Arab Emirates (UAE) University, Al Ain, UAE

**Waqar Ahmad, PhD:** [waqar.ahmad@uaeu.ac.ae](mailto:waqar.ahmad@uaeu.ac.ae)

**ORCID:** 0000-0003-0474-6558

**Neena Gopinathan Panicker, PhD:** [201990158@uaeu.ac.ae](mailto:201990158@uaeu.ac.ae)

**ORCID:** 0000-0002-7553-9006

**Tahir A. Rizvi,** **PhD:**  [tarizvi@uaeu.ac.ae](mailto:tarizvi@uaeu.ac.ae)

**ORCID:** 0000-0002-2572-1678

**Farah Mustafa, PhD:** [fmustafa@uaeu.ac.ae](mailto:fmustafa@uaeu.ac.ae)

**ORCID:** 0000-0002-1081-3756

*These authors contributed equally to this study.

^§^**Corresponding Authors:**

**Farah Mustafa, PhD.** Department of Biochemistry & Molecular Biology, Phone: +971-3-713-7509; Fax: +971-3-767-2033; E-mail: [fmustafa@uaeu.ac.ae](mailto:fmustafa@uaeu.ac.ae).

**Tahir A. Rizvi, PhD.** Department of Microbiology & Immunology, Phone: +971-3-713-7627; Fax: +971-3-767-1966; E-mail: [tarizvi@uaeu.ac.ae](mailto:tarizvi@uaeu.ac.ae).

College of Medicine and Health Sciences (CMHS), Tawam Hospital Complex, UAE University, P.O. Box 15551, Al Ain, UAE

**Running Title:** Transcriptomics of Mammary Epithelial Cells upon Differentiation

**
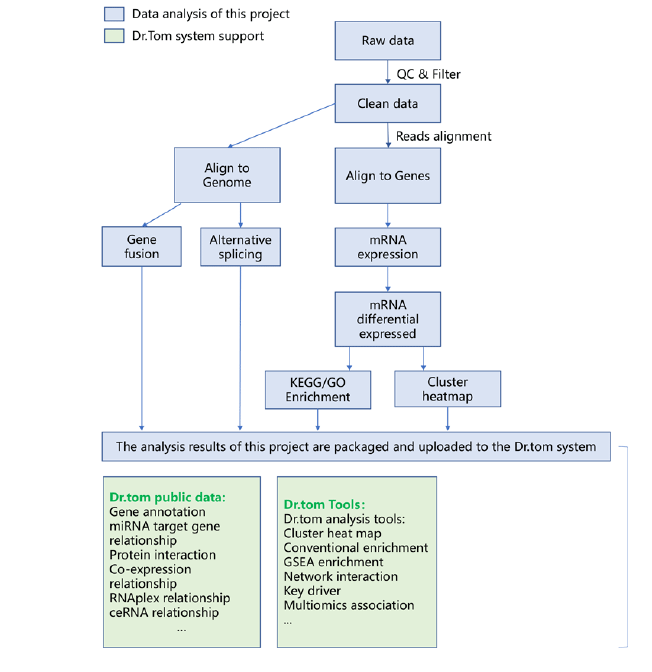
**

**Figure S1: Overview of the RNAseq data analysis pipeline performed using the BGI Dr. Tom platform.**

Raw sequencing data were subjected to quality control (QC) and filtering to generate high-quality clean reads. Clean reads were aligned to the reference genome for detection of gene fusion and alternative splicing events, and to annotated gene models for transcript-level quantification. Differential expression analysis was conducted at the mRNA level, followed by downstream functional analyses including KEGG/GO enrichment and hierarchical clustering. The resulting datasets were integrated and uploaded to the Dr. Tom system for comprehensive annotation and network-based analyses. Public annotation resources within Dr. Tom include gene annotation, miRNA–target relationships, protein interaction networks, co-expression analyses, RNAplex and ceRNA relationships. Analytical tools available through the platform include cluster heatmaps, conventional enrichment analysis, GSEA enrichment, network interaction mapping, key driver analysis, and multi-omics association modules. Blue boxes represent project-specific analyses, while green boxes indicate Dr. Tom system-supported annotation and analytical modules.

| [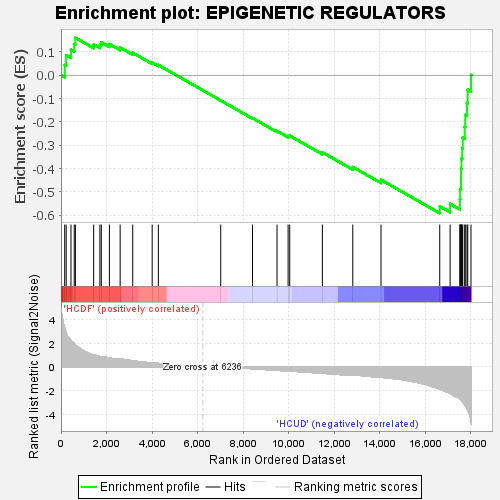](file:///C:\Users\waqar\gsea_home\output\sep21\HCDF_versus_HCUD.Gsea.1632206243665\EPIGENETIC%20REGULATORS.html) | [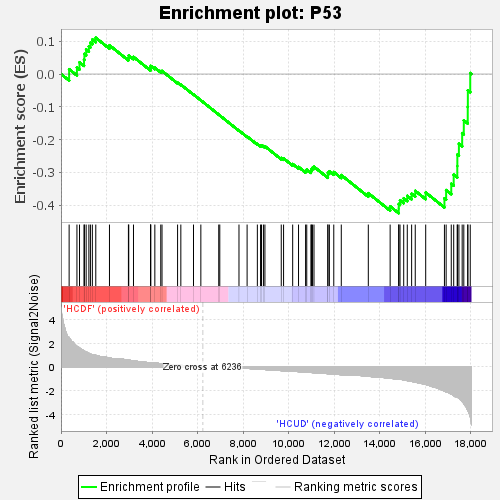](file:///C:\Users\waqar\gsea_home\output\sep21\HCDF_versus_HCUD.Gsea.1632206243665\P53.html) | [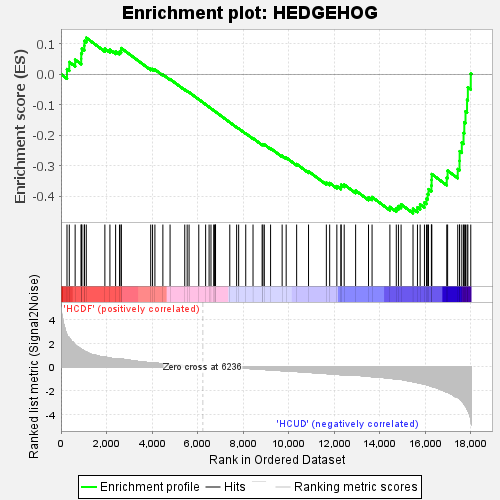](file:///C:\Users\waqar\gsea_home\output\sep21\HCDF_versus_HCUD.Gsea.1632206243665\HEDGEHOG.html) |
| --- | --- | --- |
| [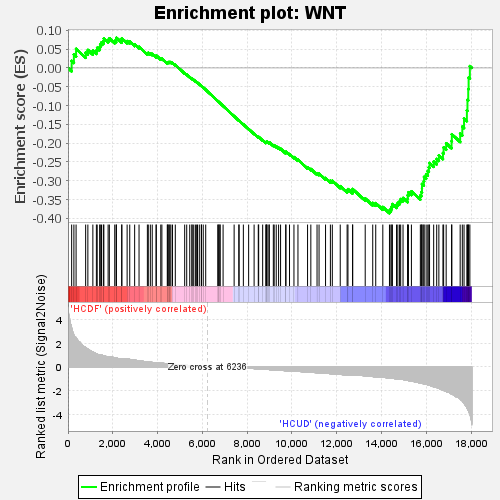](file:///C:\Users\waqar\gsea_home\output\sep21\HCDF_versus_HCUD.Gsea.1632206243665\WNT.html) | [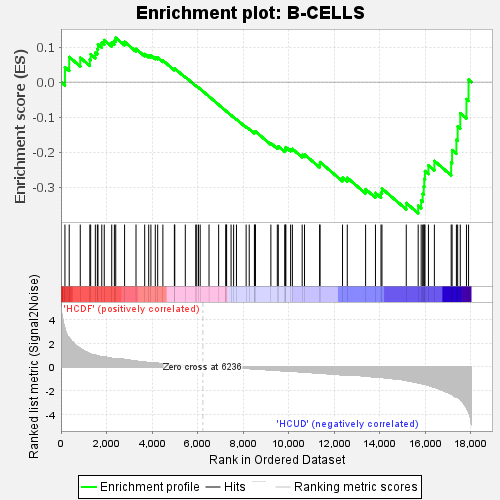](file:///C:\Users\waqar\gsea_home\output\sep21\HCDF_versus_HCUD.Gsea.1632206243665\B-CELLS.html) | [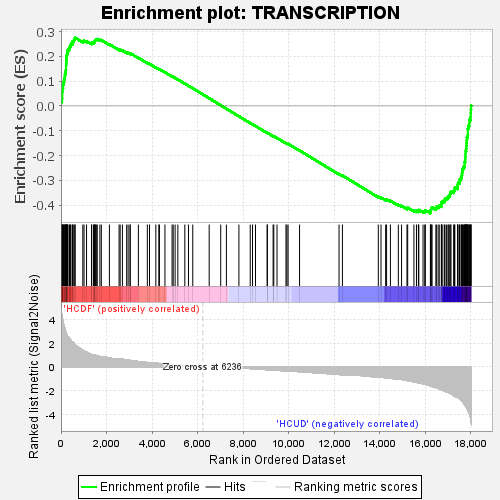](file:///C:\Users\waqar\gsea_home\output\sep21\HCDF_versus_HCUD.Gsea.1632206243665\TRANSCRIPTION.html) |
| [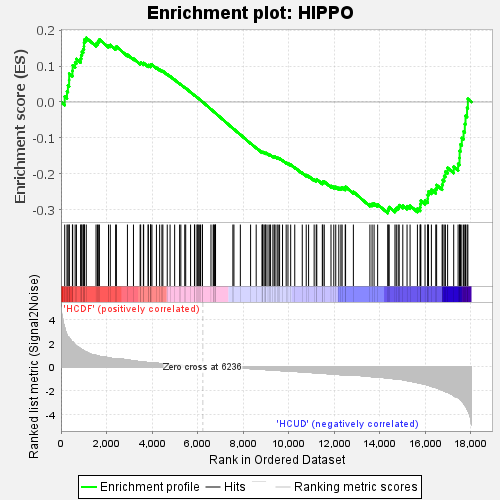](file:///C:\Users\waqar\gsea_home\output\sep21\HCDF_versus_HCUD.Gsea.1632206243665\HIPPO.html) | [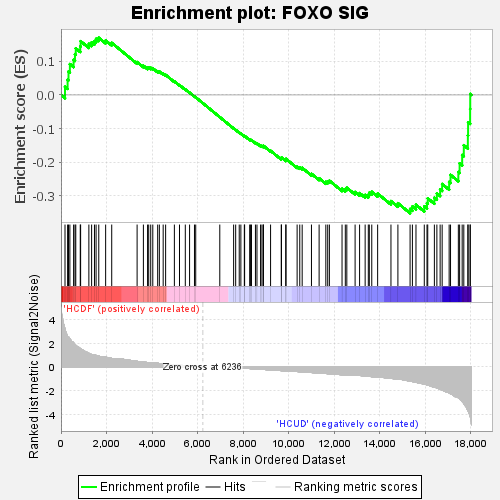](file:///C:\Users\waqar\gsea_home\output\sep21\HCDF_versus_HCUD.Gsea.1632206243665\FOXO%20SIG.html) | [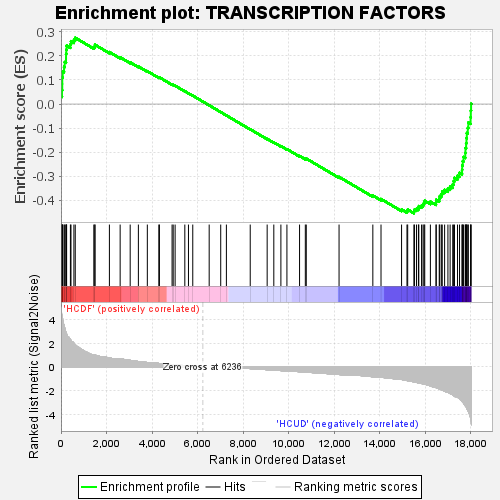](file:///C:\Users\waqar\gsea_home\output\sep21\HCDF_versus_HCUD.Gsea.1632206243665\TRANSCRIPTION%20FACTORS.html) |
| [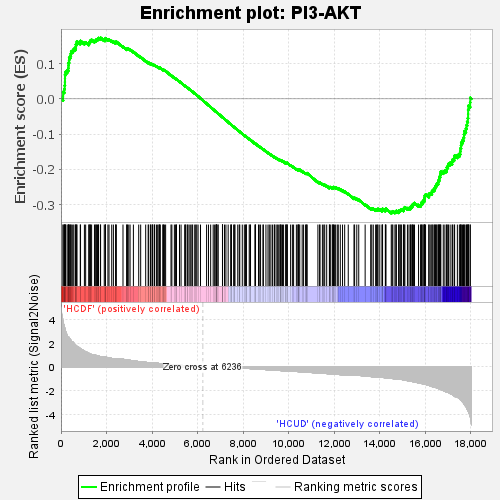](file:///C:\Users\waqar\gsea_home\output\sep21\HCDF_versus_HCUD.Gsea.1632206243665\PI3-AKT.html) | [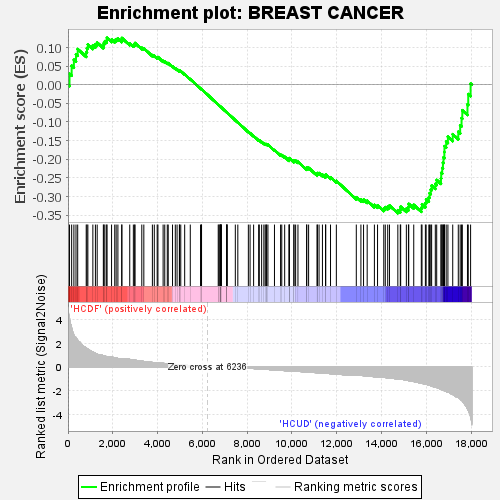](file:///C:\Users\waqar\gsea_home\output\sep21\HCDF_versus_HCUD.Gsea.1632206243665\BREAST%20CANCER.html) | [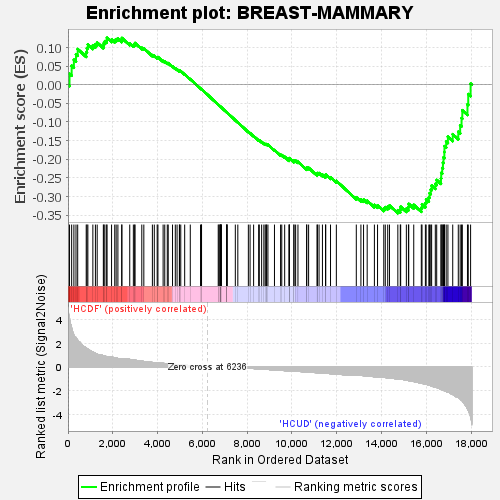](file:///C:\Users\waqar\gsea_home\output\sep21\HCDF_versus_HCUD.Gsea.1632206243665\BREAST-MAMMARY.html) |
| [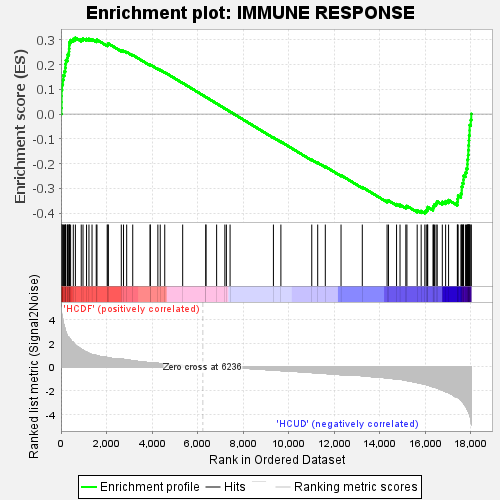](file:///C:\Users\waqar\gsea_home\output\sep21\HCDF_versus_HCUD.Gsea.1632206243665\IMMUNE%20RESPONSE.html) | [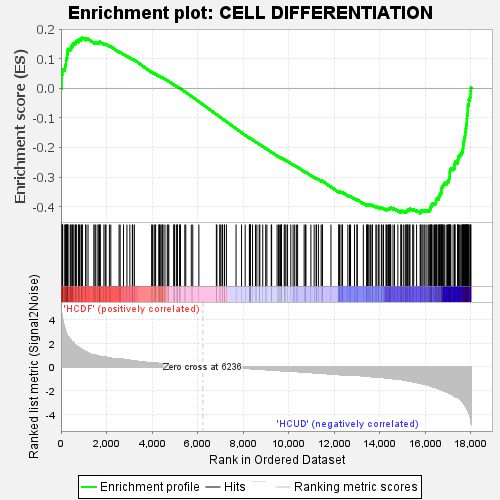](file:///C:\Users\waqar\gsea_home\output\sep21\HCDF_versus_HCUD.Gsea.1632206243665\CELL%20DIFFERENTIATION.html) | [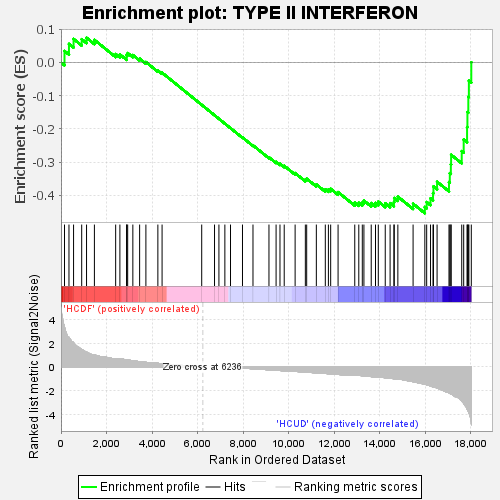](file:///C:\Users\waqar\gsea_home\output\sep21\HCDF_versus_HCUD.Gsea.1632206243665\TYPE%20II%20INTERFERON.html) |
| [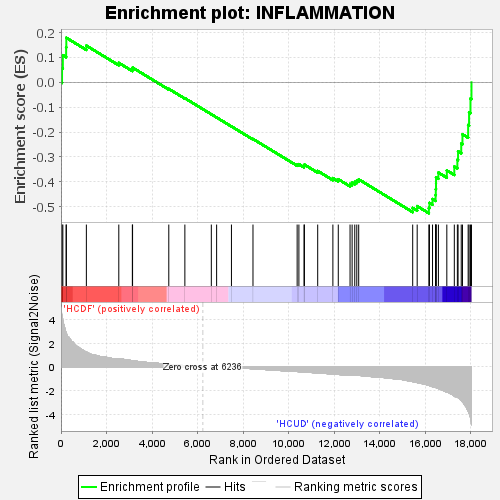](file:///C:\Users\waqar\gsea_home\output\sep21\HCDF_versus_HCUD.Gsea.1632206243665\INFLAMMATION.html) | [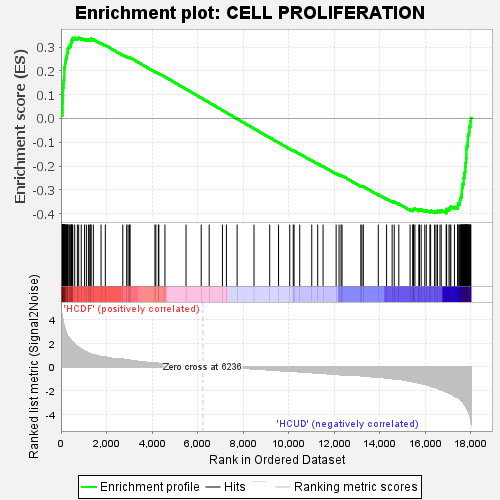](file:///C:\Users\waqar\gsea_home\output\sep21\HCDF_versus_HCUD.Gsea.1632206243665\CELL%20PROLIFERATION.html) | [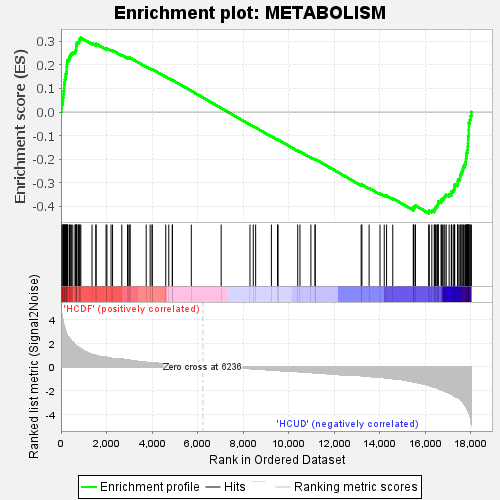](file:///C:\Users\waqar\gsea_home\output\sep21\HCDF_versus_HCUD.Gsea.1632206243665\METABOLISM.html) |
| [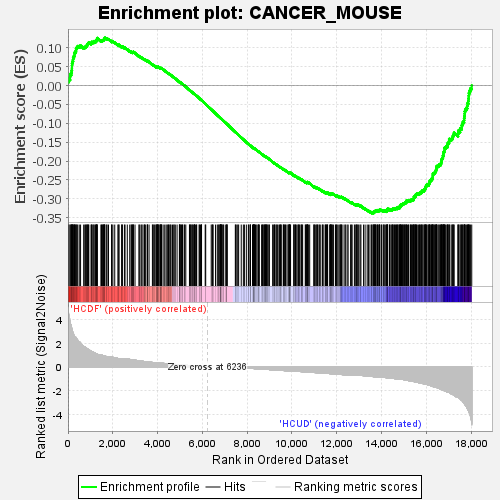](file:///C:\Users\waqar\gsea_home\output\sep21\HCDF_versus_HCUD.Gsea.1632206243665\CANCER_MOUSE.html) | [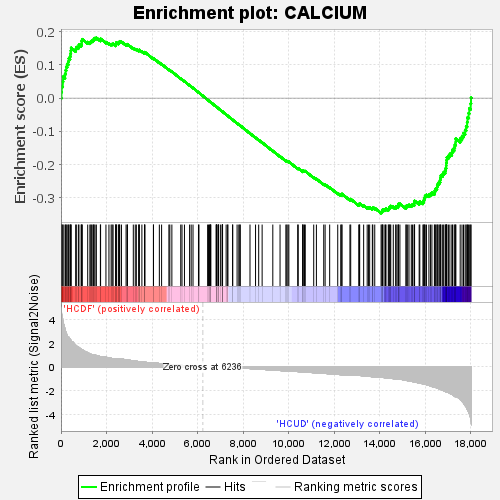](file:///C:\Users\waqar\gsea_home\output\sep21\HCDF_versus_HCUD.Gsea.1632206243665\CALCIUM.html) |  |

**Figure S2: GSEA enrichment plots for negatively enriched KEGG pathways in differentiated HC11 cells.**

Normalized enrichment plots generated by Gene Set Enrichment Analysis (GSEA) for the top negatively enriched KEGG pathways identified in the differentiated (DIFF) phenotype relative to control (CTRL). Each plot displays the enrichment score (ES) across the ranked gene list, the distribution of pathway-associated genes (black vertical bars), and the ranked metric scores. Negative normalized enrichment scores (NES) indicate coordinated transcriptional reduction of pathway-associated genes in the DIFF condition. Significance thresholds were defined as FDR *q*-value < 0.25 and FWER *p*-value < 0.2. It should be noted that GSEA reflects coordinated transcriptional changes within gene sets and does not directly measure functional pathway activity.
